# Synthesis and stability study of a new major metabolite of γ-hydroxybutyric acid

**DOI:** 10.3762/bjoc.9.72

**Published:** 2013-04-02

**Authors:** Ida Nymann Petersen, Jesper Langgaard Kristensen, Christian Tortzen, Torben Breindahl, Daniel Sejer Pedersen

**Affiliations:** 1 Department of Drug Design and Pharmacology, University of Copenhagen, Universitetsparken 2, DK-2100 Copenhagen, Denmark; 2 Department of Chemistry, University of Copenhagen, Universitetsparken 5, DK-2100 Copenhagen, Denmark; 3 Department of Clinical Biochemistry, Vendsyssel Hospital, Bispensgade 37, DK-9800 Hjørring, Denmark

**Keywords:** analytical chemistry, cabohydrate chemistry, forensic chemistry, glucuronide, γ-hydroxybutyric acid, metabolite

## Abstract

γ-Hydroxybutanoic acid (GHB) is used as a date-rape drug, which renders the victims unconscious and defenceless. Intoxications are very difficult to detect for forensic scientists due to rapid metabolism to endogenous levels of GHB. We recently discovered a new major metabolite, **2**, of GHB (**1**) that could potentially extend the analytical detection window for GHB intoxications. Herein we disclose synthetic procedures based on a Koenigs–Knorr glucuronidation approach that provides GHB glucuronide **2** and a deuterium-labelled analogue *d*_4_-**2** of high purity suitable for analytical chemistry. In addition, we have assessed the stability of GHB glucuronide **2** by mimicking the natural pH range for urine, which is of importance in the development of new analytical methods. Using NMR we show that GHB glucuronide **2** is highly stable towards aqueous hydrolysis within the pH range normally observed for urine even at elevated temperature.

## Introduction

**Figure 1:**
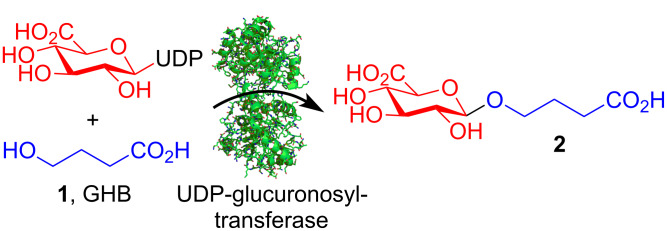
Hypothesised glucuronidation of GHB (**1**) by UDP-glucuronosyltransferase to give glucuronide **2**. UDP = Uridinediphosphate.

The abuse of illicit drugs continues to be a very significant problem to society and results in many drug-related accidents and deaths worldwide. Law enforcement agencies require the assistance of analytical laboratories to identify drugs from a wide variety of sources in order to try and combat this problem. Despite huge advances in analytical sciences certain illegal drugs continue to elude analytical detection. γ-Hydroxybutanoic acid (GHB, **1**, Figure 1), often referred to as Fantasy or liquid ecstasy, is a so-called predatory drug or date-rape drug.

Most commonly, the ingestion of GHB renders the victim unconscious and defenceless due to the heavy sedative effect, and GHB often induces short-term memory loss in victims thereby complicating case prosecution. GHB is also frequently used as a recreational drug [1] with a high risk of fatal overdosing and with a high incidence of toxic effects, including impaired consciousness, coma and numerous reports on acute poisonings and drug-related deaths [2]. After consumption, GHB is rapidly metabolised in vivo and is only detectable above endogenous levels in a narrow time window of 3–6 h [3–4]. Less than 1% of GHB is excreted unchanged in urine, and current analytical methods for serum or urine continue to be problematic. Confirmed positive laboratory samples for GHB intoxications are relatively rare, either due to delayed sampling or simply because samples are not forwarded to a toxicology laboratory [2]. Consequently any analytical method that could extend the analytical detection window for GHB would represent a very important advance in analytical and forensic science with immediate implications for society.

UDP-glucuronosyltransferase is an important enzyme in the metabolism of xenobiotics that transforms functional groups such as alcohols and carboxylic acids to their respective glucuronides (e.g., Figure 1). Glucuronides generally have longer plasma half-life values than the unmodified compound (e.g., ethyl glucuronide versus ethanol), making it possible to use the glucuronide as a biomarker to extend the analytical detection window [5]. By analogy with ethanol, we hypothesised the existence of a GHB glucuronide, and recently discovered that GHB glucuronide **2** is indeed a major metabolite of GHB (Figure 1) [6]. The presence of GHB glucuronide **2** is likely to have important implications for future analysis of GHB in clinical and forensic toxicology. The mono-sodium salt of GHB glucuronide **2** made by chemical synthesis is commercially available from Reseachem (http://www.reseachem.ch), but an isotope-labelled analogue is not available. To the best of our knowledge the synthesis or use of compound **2** has never been reported.

Herein we wish to disclose the synthesis of GHB glucuronide **2** and a deuterium labelled analogue *d*_4_-**2**, which is required as an internal standard for chromatography. Moreover, we have assessed the stability of GHB glucuronide **2** towards aqueous hydrolysis within the pH range normally observed for urine, which is of importance in the development of new analytical methods.

## Results and Discussion

### Synthesis and stability assessment

#### Synthesis of GHB glucuronides **2** and *d_4_*-**2**

**Scheme 1:**
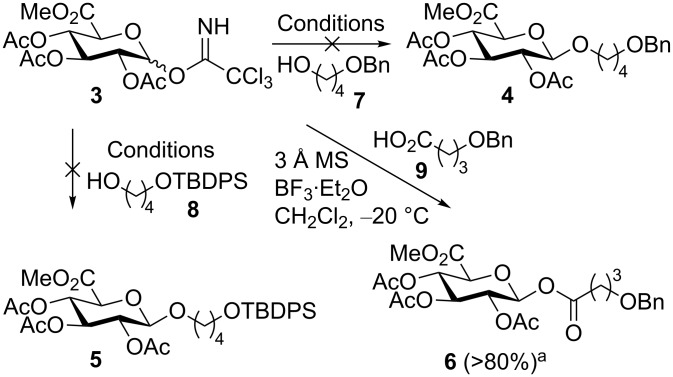
Schmidt glucuronidation [11] with trichloroacetimidate **3**. Synthesis of **4** and **5** using acceptors **7** and **8** was attempted several times by using BF_3_·OEt_2_, 3 Å MS, CH_2_Cl_2_, −20 °C to rt, and TMSOTf, 3 Å MS, CH_2_Cl_2_, −20 °C to rt, but never gave any of the desired material. ^a^Conversion to **6** with acceptor **9** was judged to be >80% by ^1^H NMR analysis of the crude product after work-up. TBDPS: *tert*-butyldiphenylsilyl; MS: molecular sieves.

The synthesis of small molecule glucuronide derivatives can be carried out by a wide variety of synthetic [7–8] and biocatalytic [9–10] methods. Initially, we favoured a synthetic approach using Schmidt trichloroacetimidate chemistry [11] with trichloroacetimidate donor **3** (Scheme 1) that has been used successfully by others for the synthesis of alcohol glucuronides [7–8,12]. Moreover, the required trichloroacetimidate donor **3** is stable and accessible from commercially available glucuronolactone by using literature methods (Scheme 1) [13–16]. We anticipated that glucuronidation with a mono-protected 1,4-butanediol acceptor [17–19] would be feasible and that it would be possible to deprotect and oxidise the glucuronidation product (**4** or **5**) to provide target molecule **2**.

**Scheme 2:**
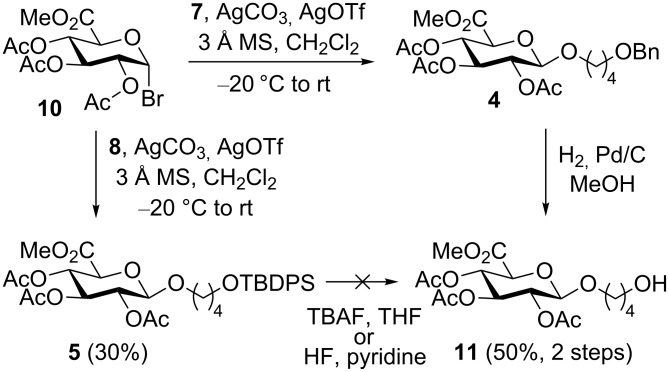
Koenigs–Knorr glucuronidation [27] with bromide **10** and acceptors **7** and **8**.

However, attempts under commonly employed reaction conditions for glucuronidation returned none of the desired product **4** or **5**. Glucuronidation of alcohols with trichloroacetimidate **3** has been reported to be problematic due to the high reactivity of the acceptor relative to the donor resulting in trans-esterification [20–23]. Indeed in our case acetylated acceptor was the only identified product from the reaction. To evaluate whether the high reactivity of the acceptor was the problem we tested the less reactive acceptor 4-benzyloxybutanoic acid (**9**). As anticipated a less reactive acceptor provided the glucuronidated product **6** in high yield as estimated by ^1^H NMR on the crude reaction mixture. Trans-esterification during glucuronidation can be suppressed by changing from acetyl protection on the sugar moiety to less reactive benzoyl, isobutyroyl or pivaloyl protection groups [21–23]. Alternatively, the use of bromo-derivative **10** (Scheme 2), which is easily synthesised in two steps from glucuronolactone [14,24] has been shown to glucuronidate primary and secondary alcohols under Koenigs–Knorr conditions [7–8,25–26].

**Scheme 3:**
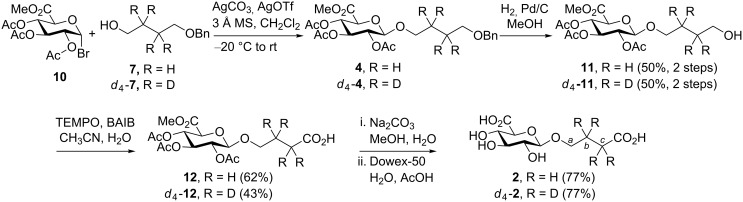
Synthesis of GHB glucuronides **2** and *d*_4_-**2** by using a Koenigs–Knorr glucuronidation approach. TEMPO: 2,2,6,6-tetramethyl-1-piperidinyloxyl, BAIB: [bis(acetoxy)iodo]benzene.

Due to the easy access of donor **10** from glucuronolactone we decided to explore the Koenigs–Knorr glucuronidation route [27]. Using standard Koenigs–Knorr conditions donor **10** does indeed glucuronidate acceptor **8** to give the desired product **5** albeit only in 30% yield. Unfortunately, removal of the TBDPS protection group to provide the desired alcohol **11** proved difficult and complex mixtures were obtained on using both TBAF in THF and HF in pyridine. Fortunately, glucuronidation also proceeded with acceptor **7** to give **4**, and in this case the benzyl group was easily removed by catalytic hydrogenation to provide alcohol **11** in good yield. Oxidation of alcohol **11** was carried out similarly to that reported elsewhere [19], using Epp and Widlanski’s TEMPO oxidation procedure [28] to furnish carboxylic acids **12** and *d*_4_-**12** (Scheme 3). Finally, deprotection under basic condition followed by treatment with an acidic ion-exchange resin provided the required GHB glucuronides **2** and *d*_4_-**2** in good yield.

**Figure 2:**
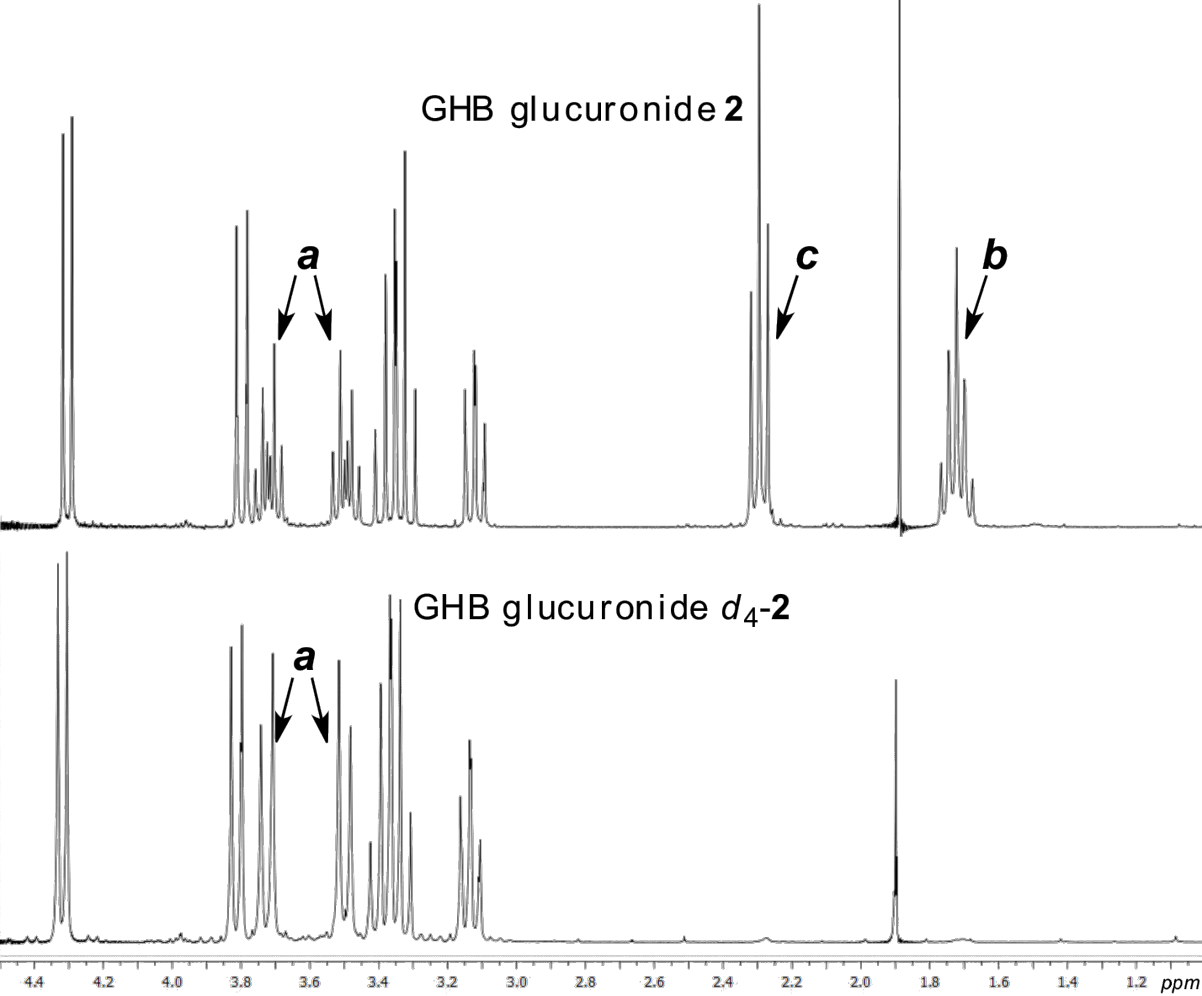
^1^H NMR spectrum (D_2_O, 300 MHz) of GHB glucuronides **2** (top) and *d*_4_-**2** (bottom). As anticipated, methylene protons **b** and **c** are absent in *d*_4_-**2** (cf. labelling in Scheme 3).

^1^H NMR analysis of *d*_4_-**2** showed the complete absence of methylene groups **b** and **c** (Figure 2). In addition, analysis of *d*_4_-**2** by mass spectrometry showed the presence of less than 0.14% of **2**, thus satisfying the demand for a highly pure internal standard [6].

#### Stability assessment of GHB glucuronide **2** by NMR

**Figure 3:**
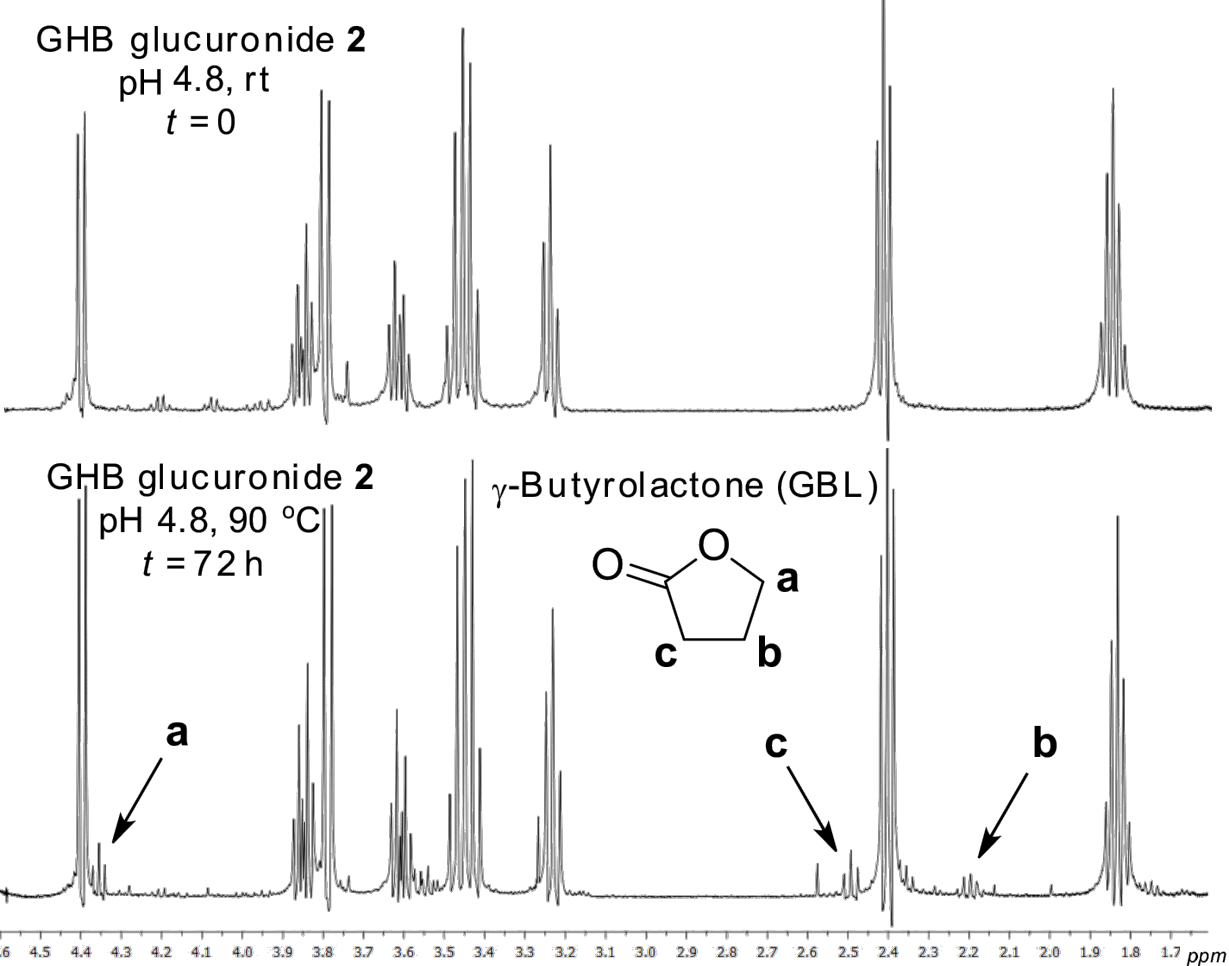
^1^H NMR spectra (500 MHz) of GHB glucuronide **2** in pH 4.8 buffer at *t* = 0 (rt) and *t* = 72 h (90 °C) by using a Watergate-type water suppression method (Supporting Information File 1). After heating at 90 °C for 72 h GBL starts to form at low concentration (indicated with arrows).

The stability of GHB glucuronide **2** is critical if it is to be used for routine analysis by analytical and forensic chemists. Consequently, a series of NMR experiments to assess the stability of GHB glucuronide **2** were conducted. To mimic the normal pH range for urine (pH 4.6–8) mono- and a di-basic sodium phosphate buffers were employed as NMR solvents to give pH values of 4.8 and 9.0, respectively (Supporting Information File 1). The stability of GHB glucuronide **2** was assessed from 18 to 90 °C for several days. GHB glucuronide **2** was found to be almost completely stable in both buffer systems over the entire temperature range. Only after heating at 90 °C in acidic buffer for 3 days could a small amount of γ-butyrolactone (GBL) be detected (Figure 3). Under forcing acidic conditions (autoclaving for 15 min with 4 M aq HCl) GHB glucuronide **2** was completely degraded whilst being stable towards strong base (3 M aq NaOH) [6].

## Conclusion

Herein we have described the synthesis of a recently discovered major metabolite of GHB that has the potential to extend the analytical detection window for GHB intoxications significantly. GHB glucuronide **2** and the isotope-labelled analogue *d*_4_-**2** were shown to be of sufficient purity for use in analytical laboratories. Moreover, the stability of GHB glucuronide **2** was assessed under basic and acidic conditions mimicking the pH range typically observed in urine samples. GHB glucuronide was demonstrated to be highly stabile towards aqueous hydrolysis within the pH range normally observed for urine even at elevated temperature for several days, making it suitable for method development within analytical and forensic chemistry.

## Experimental

### General

For reactions conducted under anhydrous conditions, glassware was dried overnight in an oven at 150 °C and was allowed to cool in a desiccator over anhydrous KOH. Anhydrous reactions were carried out under nitrogen. THF was distilled from sodium wire with benzophenone as indicator. Dichloromethane and pyridine were dried and stored over 4 Å molecular sieves. Thin-layer chromatography (TLC) was carried out on commercially available precoated aluminium sheets (Merck 60 F_254_). The quoted *R*_f_ values are rounded to the nearest 0.05. ^1^H and ^13^C NMR was run on a Varian Mercury 300 MHz, a Varian Gemini 300 MHz and a Bruker 500 MHz Avance III Fourier transform NMR spectrometer, respectively, by using an internal deuterium lock. Solvents were used as internal standard when assigning NMR spectra [29]. *J* values are given in hertz (Hz) and rounded to the nearest 0.5 Hz. Dry column vacuum chromatography (DCVC) was carried out according to the published procedure [30]. High-resolution mass spectra were recorded on a Micromass Q-TOF 1.5, UB137. Melting points were recorded on an OptiMelt MPA100 from Stanford Research Systems.

Glucuronide donors **10** [24] and **3** [13–16] and acceptors **8**, **7** and *d*_4_-**7** [17–19] were synthesised according to literature procedures. All analytical data were in agreement with those previously published.

**Methyl 2,3,4-tri-*O*-acetyl-1-*O*-(1-hydroxybut-4-yl)-β-D-glucopyranosiduronate (11):** Bromide **10** (0.6 g, 1.17 mmol) and 4-benzyloxybutan-1-ol (**7**) (0.3 g, 1.17 mmol) were dissolved in anhydrous CH_2_Cl_2_ (10 mL) and stirred with molecular sieves (3 Å) for 1 h. The reaction mixture was cooled to −20 °C before AgOTf (0.43 g, 1.17 mmol) and Ag_2_CO_3_ (0.46 g, 1.17 mmol) were added. The mixture was stirred for 3 h at −20 °C and the solids were removed by filtration through a pad of Celite. Sat. aq NaHCO_3_ solution (50 mL) was added_,_ and the mixture was extracted with EtOAc (3 × 40 mL). The combined organic phases were dried (MgSO_4_), filtered and evaporated in vacuo. The residue was purified by DCVC [id 2 cm; 20 mL fractions 20% EtOAc in *n*-heptane (100 mL) (v/v); 50% EtOAc in *n*-heptane (100 mL) (v/v)] to give glucuronide **4** (0.48 g) contaminated with bromide **10** and alcohol **7**. With no further purification the mixture was dissolved in MeOH (19 mL) and Pd on activated charcoal (10% w/w, 20 mg) was added, and then the flask was fitted with a H_2_ balloon and stirred vigorously. After 24 h the mixture was filtered through a pad of Celite and concentrated in vacuo. The residue was purified by DCVC [id 4 cm; 20 mL fractions 20% EtOAc in *n*-heptane (100 mL) (v/v); 80% EtOAc in *n*-heptane (100 mL) (v/v)] to give glucuronide **11** (0.16 g, 50%) as colourless plates. Mp: 78.4 °C (from EtOAc, *n*-heptane); 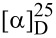 −80.0 (*c* 0.5, MeOH); *R*_f_ 0.3 (80% EtOAc in *n*-heptane, v/v); IR (CHCl_3_) ν_max_: 3300 (OH), 1724 (C=O) cm^−1^; MS (ESI^+^) *m/z*: [M + Na]^+^ calcd for C_17_H_26_O_11_Na, 429.1373; found, 429.1392; ^1^H NMR (300 MHz, CDCl_3_) δ 5.29–5.17 (m, 2H, H3 and H4), 5.06–4.46 (m, 1H, H2), 4.50 (d, *J* = 8 Hz, 1H, H1), 4.05 (m, 1H, H4), 3.97 (m, 1H, H6), 3.76 (s, 3H, Me), 3.66 (q, *J* = 6 Hz, 2H, H9), 3.56 (m, 1H, H6), 2.07 (s, 3H, Ac), 2.04 (2 s, 6H, 2 × Ac), 1.65 (m, 4H, H7 and H8); ^13^C NMR (300 MHz, CDCl_3_) δ 170.1, 169.4, 169.3, 167.2 (4 × C=O), 100.8 (C1), 72.7, 72.1, 71.4, 70.3, 69.5 (4 × CH and 1 × CH_2_), 62.5 (CH_2_OH), 53.1 (CO_2_*C*H_3_), 29.4, 26.0 (2 × CH_2_), 20.9 (2 × Ac), 20.8 (Ac).

**Methyl 2,3,4-tri-*O*-acetyl-1-*O*-(1-carboxyprop-3-yl)-β-D-glucopyranosiduronate (12):** Alcohol **11** (0.16 g, 0.4 mmol), 2,2,6,6-tetramethylpiperinyloxyl (12.5 mg, 0.08 mmol) and [bis(acetoxy)iodo]benzene (0.28 g, 0.88 mmol) were dissolved in H_2_O/CH_3_CN (1 mL). After 12 h water (20 mL) was added and the mixture was extracted with EtOAc (2 × 20 mL). The combined organic phases were washed with water (40 mL), dried (MgSO_4_) and evaporated in vacuo. The residue was purified by DCVC [id 2 cm; 20 mL fractions 25% EtOAc in *n-*heptane (100 mL); 75% EtOAc in *n-*heptane (100 mL) (v/v)] to give carboxylic acid **12** (0.10 g, 62%) as colourless needles. 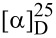 −22.0 (*c* 1, MeOH); IR (CHCl_3_) ν_max_: 3399 (O-H) and 1754 (C=O) cm^−1^; MS (ESI^+^) *m/z*: [M + Na]^+^ calcd for C_17_H_24_O_12_Na, 443.1165; found, 443.1181; ^1^H NMR (300 MHz, CDCl_3_) δ 5.29–5.17 (m, 2H, H3 and H4), 5.06–4.46 (m, 1H, H2), 4.5 (d, *J* = 8 Hz, 1H, H1), 4.05 (m, 1H, H5), 3.97 (m, 1H, H6), 3.76 (s, 3H, Me), 3.56 (m, 1H, H6), 2.45 (t, *J* = 7 Hz, 2H, H8), 2.07 (s, 3H, Ac), 2.04 (2 s, 6H, 2 × Ac), 1.93 (m, 2H, H7); ^13^C NMR (300 MHz, CDCl_3_) δ 178.7 (CO_2_H), 170.1, 169.4, 169.3, 167.2 (4 × C=O), 100.8 (C1), 72.7, 72.1, 71.3, 69.5, 69.0 (4 × CH and 1 × CH_2_), 53.1 (CO_2_*C*H_3_), 30.3 (CH_2_), 24.6 (CH_2_), 20.8 (2 × Ac), 20.7 (Ac).

**1-O-(3-Carboxypropyl)-β-D-glucopyranosiduronic acid (2):** Carboxylic ester **12** (0.14 g, 0.33 mmol) was dissolved in water (4 mL) and methanol (12 mL) before Na_2_CO_3_ (0.21 g, 2 mmol) was added. After 2 days water (2 mL) and glacial acetic acid (0.1 mL) were added. The mixture was filtered through a short column of Dowex-50 resin (prewashed with 3 mL MeOH), and the resin was washed with water (10 mL). The solvents were evaporated in vacuo to give carboxylic acid **2** (72 mg, 77%) as a clear gum that required no further purification. 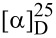 −44.0 (*c* 1, H_2_O); *R*_f_ 0.45 (1:1:1:1 EtOAc/*n*-butanol/acetic acid/water, v/v/v/v); IR (CHCl_3_) ν_max_: 3400 (O-H) and 1715 (C=O) cm^−1^; MS (ESI^+^) *m/z*: [M + Na]^+^ calcd for C_10_H_16_O_9_Na, 303.0692; found, 303.0694; ^1^H NMR (300 MHz, CDCl_3_) δ 4.31 (d, *J* = 7.5 Hz, 1H, H1), 3.8 (m, 1H, H5), 3.7 (dt, *J* = 10.0, 6.5 Hz, 1H, H6), 3.5 (dt, *J* = 10.0, 6.5 Hz, 1H, H6), 3.35 (m, 2H, H3 and H4), 3.1 (m, 1H, H2), 2.3 (t, *J =* 7.4 Hz*,* 2H, H8), 1.72 (m, 2H, H7); ^13^C NMR (300 MHz, CDCl_3_) δ 178.4, 172.3 (2 × C=O), 102.5 (C1), 75.5, 74.7, 73.0, 71.6, 69.8 (4 × CH and 1 × CH_2_), 30.6 (*C*H_2_C=O), 24.7 (*C*H_2_CH_2_C=O).

**Methyl 2,3,4-tri-*O*-acetyl-1-*O*-(2,3-[^2^H_4_]-1-hydroxybut-4-yl)-β-D-glucopyransiduronate (*d*_4_-11):** Prepared as described above to give alcohol *d*_4_-**11** (407 mg, 50%) as white needles. 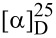 +23.0 (*c* 1, MeOH); *R*_f_ 0.25 (1:1 EtOAc/*n*-heptane, v/v); IR (CHCl_3_) ν_max_: 3399 (O-H) and 1754 (C=O) cm^−1^; MS (ESI^+^) *m/z*: [M + Na]^+^ calcd for C_17_H_22_D_4_O_11_Na, 433.1625; found, 433.1634; ^1^H NMR (300 MHz, CDCl_3_) δ 5.29–5.17 (m, 2H, H3 and H4), 5.06–4.46 (m, 1H, H2), 4.55 (d, *J* = 8 Hz, 1H, H1), 4.05 (m, 1H, H4), 3.94 (d, *J* = 10 Hz, 1H, H6), 3.76 (s, 3H, Me), 3.66 (s, 2H, H9), 3.56 (d, *J* = 10 Hz, 1H, H6), 2.07 (s, 3H, Ac), 2.04 (2 s, 6H, 2 × Ac); ^13^C NMR (300 MHz, CDCl_3_) δ 170.5, 169.7, 169.6, 167.6 (4 × C=O), 101.2 (C1), 73.0, 72.5, 71.7, 70.5, 69.9 (4 × CH and 1 × CH_2_), 62.7 (CH_2_OH), 53.4 (CO_2_*C*H_3_), 43.8 (m, *C*D_2_CH_2_OH), 25.5 (m, *C*D_2_), 21.2 (2 × Ac), 21.1 (Ac).

**Methyl 2,3,4-tri-*O*-acetyl-(1,2-[^2^H_4_]-1-carboxyprop-3-yl)-β-D-glucopyransiduronate (*d_4_*-12):** Prepared as described above to give carboxylic acid *d*_4_-**12** (140 mg, 43%) as white needles. 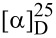 +21.8 (*c* 1, MeOH); IR (CHCl_3_) ν_max_: 3399 (O-H) and 1754 (C=O) cm^−1^; MS (ESI^+^) *m/z*: [M + Na]^+^ calcd for C_17_H_22_D_4_O_11_Na, 447.1418; found, 447.1374; ^1^H NMR (300 MHz, CDCl_3_) δ 5.29–5.17 (m, 2H, H3 and H4), 5.06–4.46 (m, 1H, H2), 4.55 (d, *J* = 8 Hz, 1H, H1), 4.05 (m, 1H, H4), 3.94 (d, *J* = 10 Hz, 1H, H6), 3.76 (s, 3H, Me), 3.66 (s, 2H, H9), 3.56 (d, *J* = 10 Hz, 1H, H6), 2.07 (s, 3H, Ac), 2.04 (2 s, 6H, 2 × Ac); ^13^C NMR (300 MHz, CDCl_3_) δ 178.8 (CO_2_H), 170.5, 169.7, 169.7, 167.5 (4 × C=O), 101.1 (C1), 73.0, 72.5, 71.6, 69.9, 69.1 (4 × CH and 1 × CH_2_), 53.4 (CO_2_*C*H_3_), 29.8 (m, *C*D_2_C=O), 24.2 (m, *C*D_2_CD_2_C=O), 20.8 (2 × Ac), 20.7 (Ac).

**1-*O*-(1,2-[^2^H_4_]-1-Carboxyprop-3-yl)-β-D-glucopyranosiduronic acid (*d_4_*-2):** Prepared as described above to give carboxylic acid *d*_4_-**2** (55 mg, 77%) as a clear colourless gum. 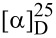 −46.0 (*c* 1, H_2_O); *R*_f_ 0.45 (1:1:1:1 EtOAc/*n*-butanol/acetic acid/water, v/v/v/v); IR (CHCl_3_) ν_max_: 3400 (O-H) and 1715 (C=O) cm^−1^; MS (ESI^+^) *m/z*: [M + Na]^+^ calcd for C_10_H_12_D_4_O_9_Na, 307.095; found, 307.0951; ^1^H NMR (300 MHz, CDCl_3_) δ 4.32 (d, *J* = 8 Hz, 1H, H1), 3.8 (m, 1H, H5), 3.7 (d, *J* = 10 Hz, 1H, H6), 3.5 (d, *J* = 10, 6.5 Hz, 1H, H6), 3.37 (m, 2H, H3 and H4), 3.1 (m, 1H, H2); ^13^C NMR (300 MHz, CDCl_3_) δ 178.3, 172.3 (2 × C=O), 102.5 (C1), 75.5, 74.7, 73.0, 71.6, 69.8 (4 × CH and 1 × CH_2_), 30.0 (m, *C*D_2_C=O), 23.8 (m, *C*D_2_CD_2_C=O).

## Supporting Information

File 1 1D and 2D NMR spectra for **2** and *d*_4_-**2** and all details for the NMR stability study of GHB glucuronide **2**.

## References

[1] Carter L P, Pardi D, Gorsline J, Griffiths R R (2009). Drug Alcohol Depend.

[2] Knudsen K, Greter J, Verdicchio M (2008). Clin Toxicol.

[3] Haller C, Thai D, Jacob P I, Dyer J E (2006). J Anal Toxicol.

[4] Brailsford A D, Cowan D A, Kicman A T (2012). J Anal Toxicol.

[5] Jatlow P, O'Malley S S (2010). Alcohol: Clin Exp Res.

[6] Petersen I N, Kristensen J L, Tortzen C, Pedersen D S, Breindahl T (2013). J Anal Toxicol.

[7] Stachulski A V, Jenkins G N (1998). Nat Prod Rep.

[8] Kaspersen F M, van Boeckel C A A (1987). Xenobiotica.

[9] Wilkinson S M, Liew C W, Mackay J P, Salleh H M, Withers S G, McLeod M D (2008). Org Lett.

[10] Khymenets O, Joglar J, Clapés P, Parella T, Covas M-I, de la Torre R (2006). Adv Synth Catal.

[11] Schmidt R R, Michel J (1980). Angew Chem, Int Ed Engl.

[12] Pews-Davtyan A, Pirojan A, Shaljyan I, Awetissjan A A, Reinke H, Vogel C (2003). J Carbohydr Chem.

[13] Brown R T, Scheinmann F, Stachulski A V (1997). J Chem Res, Synop.

[14] Bollenback G N, Long J W, Benjamin D G, Lindquist J A (1955). J Am Chem Soc.

[15] Trynda A, Madaj J, Konitz A, Wiśniewski A (2000). Carbohydr Res.

[16] Dumont-Hornebeck B A, Joly J-P, Coulon J, Chapleur Y (1999). Carbohydr Res.

[17] Djerassi C, Sheehan M, Spangler R J (1971). J Org Chem.

[18] George S, Sudalai A (2007). Tetrahedron: Asymmetry.

[19] Raunkjær M, Pedersen D S, Elsey G M, Sefton M A, Skouroumounis G K (2001). Tetrahedron Lett.

[20] Berrang B, Brine G A, Carroll F I (1997). Synthesis.

[21] Brown R T, Carter N K, Lumbard K W, Scheinmann F (1995). Tetrahedron Lett.

[22] Brown R T, Carter N E, Mayalarp S P, Scheinmann F (2000). Tetrahedron.

[23] Lucas R, Alcantara D, Morales J C (2009). Carbohydr Res.

[24] Yu H N, Furukawa J-i, Ikeda T, Wong C-H (2004). Org Lett.

[25] Agnihotri G, Misra A K (2006). Carbohydr Res.

[26] Kim H-J, Ahn K C, Ma S J, Gee S J, Hammock B D (2007). J Agric Food Chem.

[27] Koenigs W, Knorr E (1901). Ber Dtsch Chem Ges.

[28] Epp J B, Widlanski T S (1999). J Org Chem.

[29] Gottlieb H E, Kotlyar V, Nudelman A (1997). J Org Chem.

[30] Pedersen D S, Rosenbohm C (2001). Synthesis.

