# Supplementary material for: Synthesis and stability study of a new major metabolite of γ-hydroxybutyric acid
Source: Beilstein J Org Chem. 2013 Apr 2;9:641–6. doi: 10.3762/bjoc.9.72 (PMC3628683; doi:10.3762/bjoc.9.72)
Supplement: File 1 — 1D and 2D NMR spectra for 2 and d4-2 and all details for the NMR stability study of GHB glucuronide 2. [file Beilstein_J_Org_Chem-09-641-s001.pdf]

Supporting Information

for

Synthesis and stability study of a new major metabolite of γ-hydroxybutyric acid

Ida Nymann Petersen[1], Jesper Langgaard Kristensen[1], Christian Tortzen[2], Torben Breindahl[3] and Daniel Sejer Pedersen[*1]

Address: [1]Department of Drug Design and Pharmacology, University of Copenhagen, Universitetsparken 2, DK-2100 Copenhagen, Denmark, [2]Department of Chemistry, University of Copenhagen, Universitetsparken 5, DK-2100 Copenhagen, Denmark and [3]Department of Clinical Biochemistry, Vendsyssel Hospital, Bispensgade 37, DK-9800 Hjørring, Denmark

Email: Daniel Sejer Pedersen – daniel.pedersen@sund.ku.dk

* Corresponding author

1D and 2D NMR spectra for 2 and d_4-2 and all details for the NMR stability study of GHB glucuronide 2

Table of Contents

1. NMR characterisation

1.1 1D and 2D NMR spectra of glucuronide **2** S3

1.2 1D and 2D NMR spectra of glucuronide d_4-**2** S8

2. NMR stability study for GHB-GLUC (2)

2.1 General NMR experimental S13

2.2 NMR stability study ^{1}H NMR spectra S15

2.3 NMR reference spectra S18

1.1 1D and 2D NMR spectra of glucuronide 2

^{1}H NMR spectrum of GHB glucuronide 2

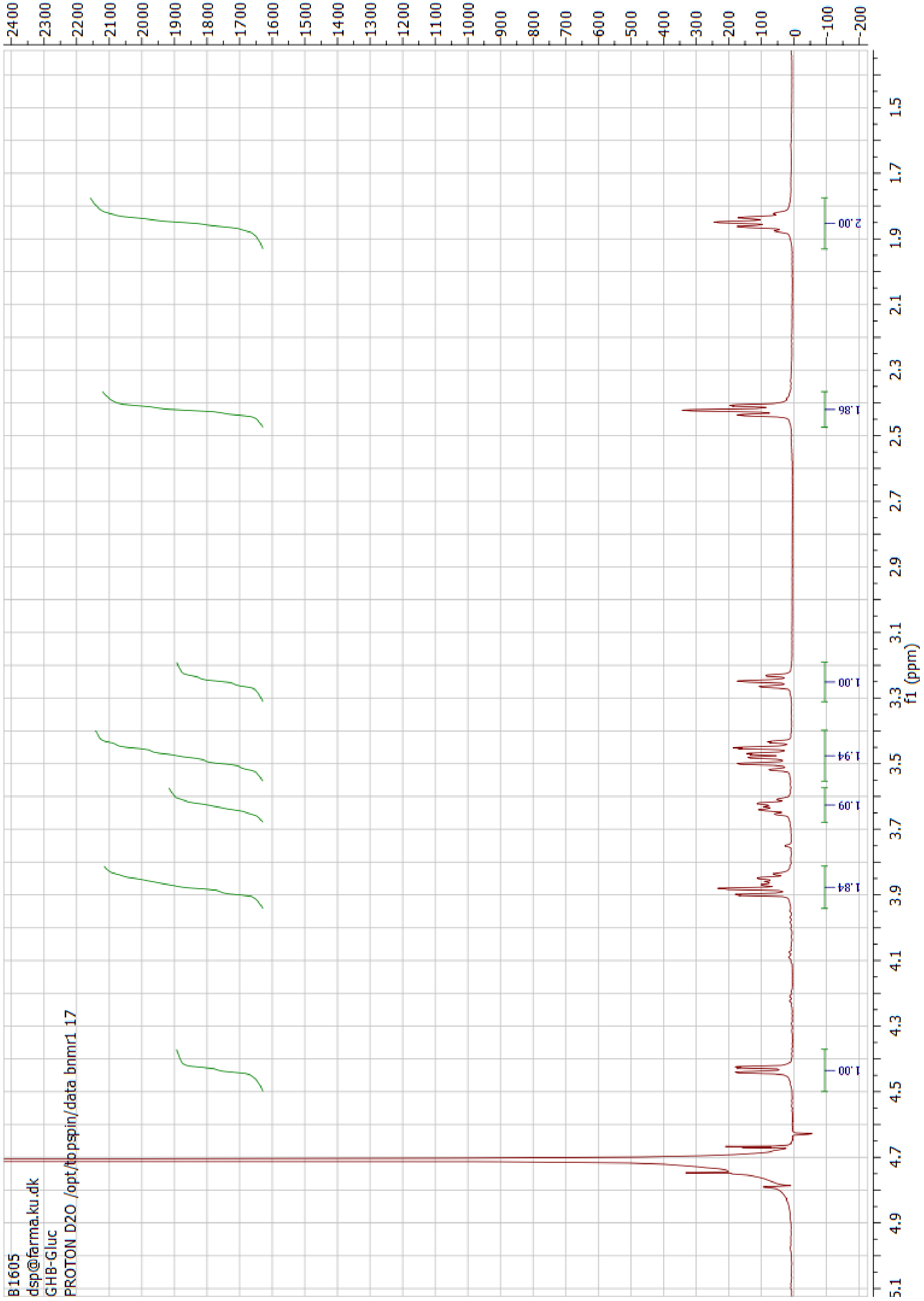

COSY NMR spectrum of GHB glucuronide 2

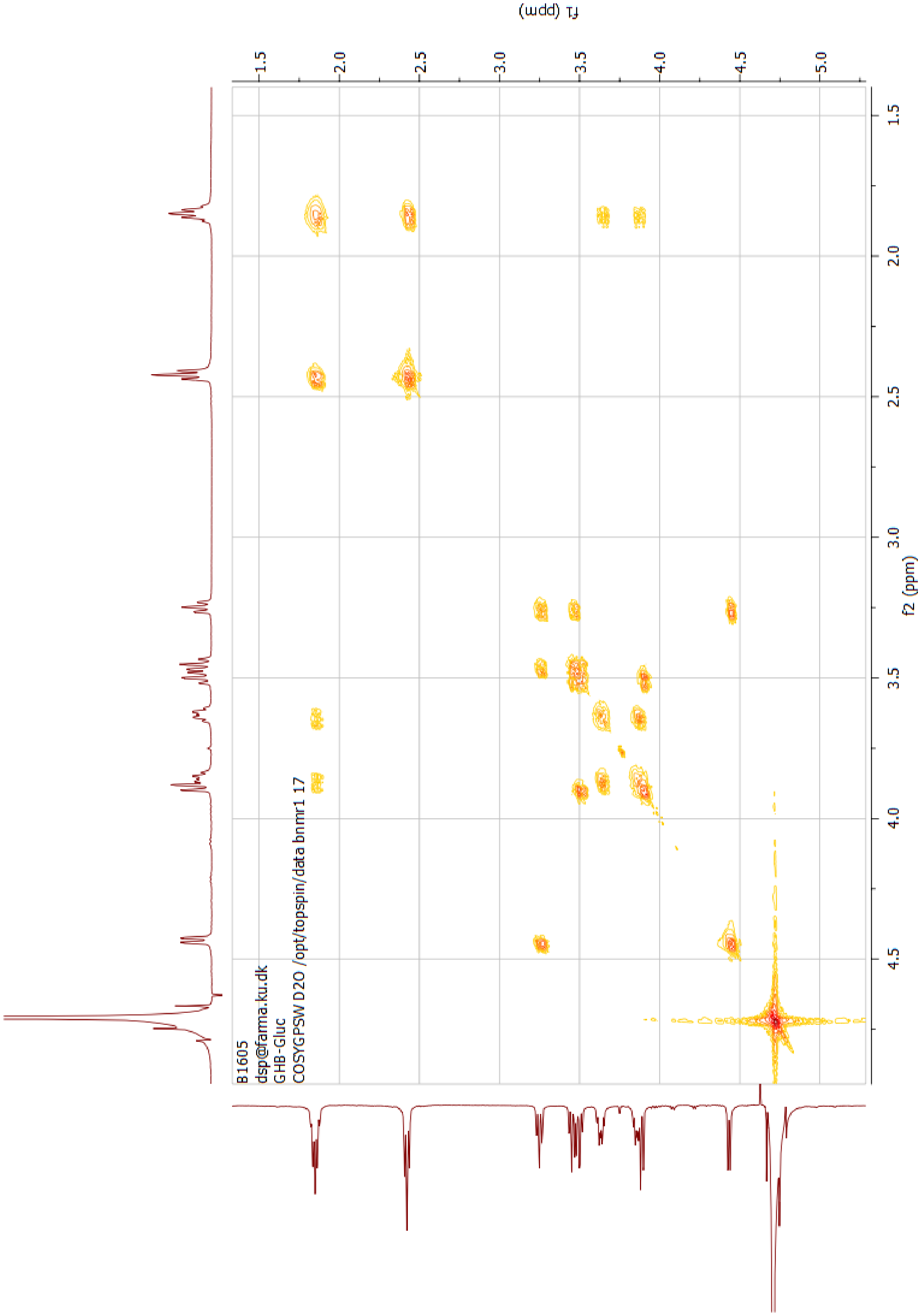

^{13}C APT NMR spectrum of GHB glucuronide 2

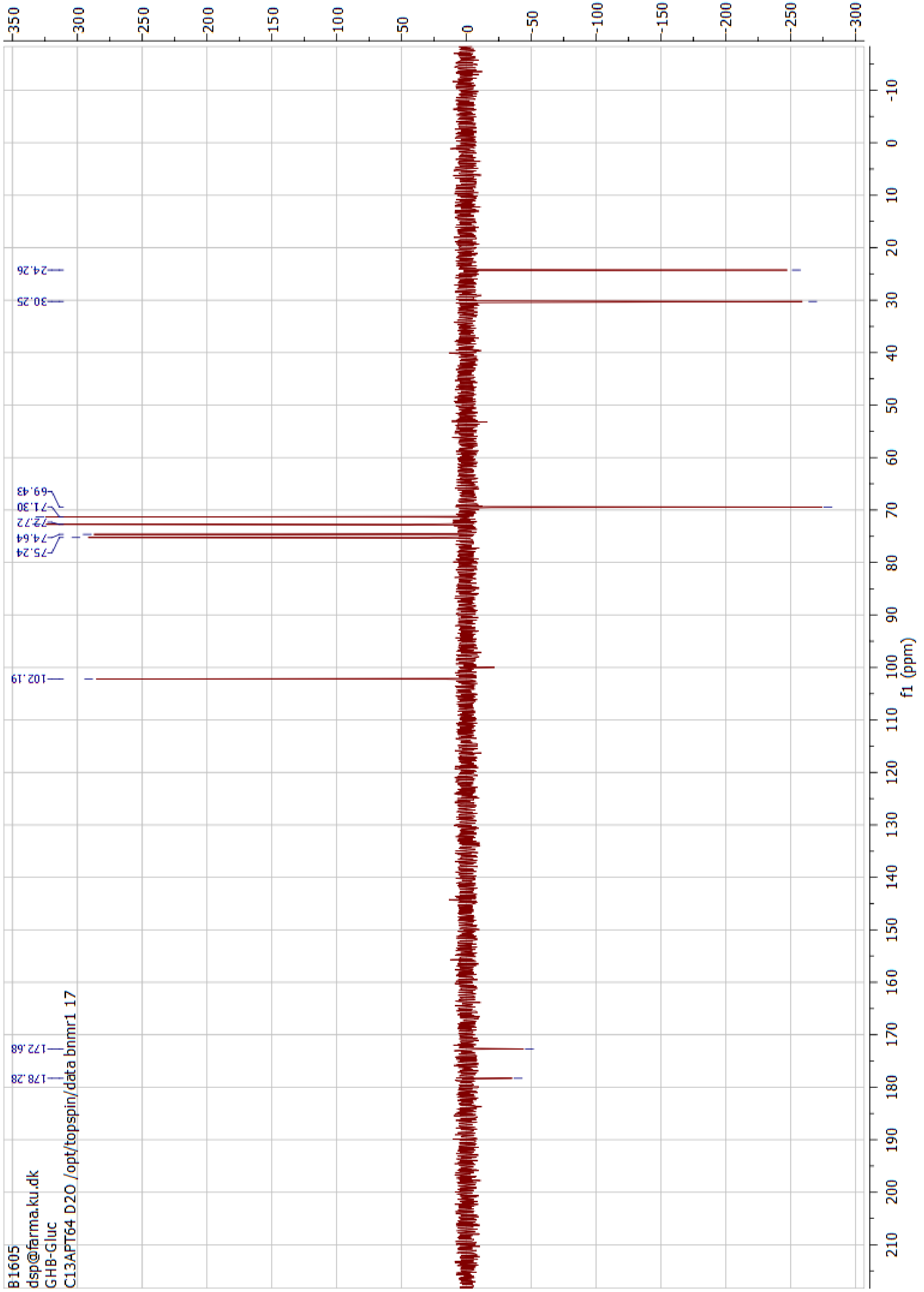

HSQC NMR spectrum of GHB glucuronide 2

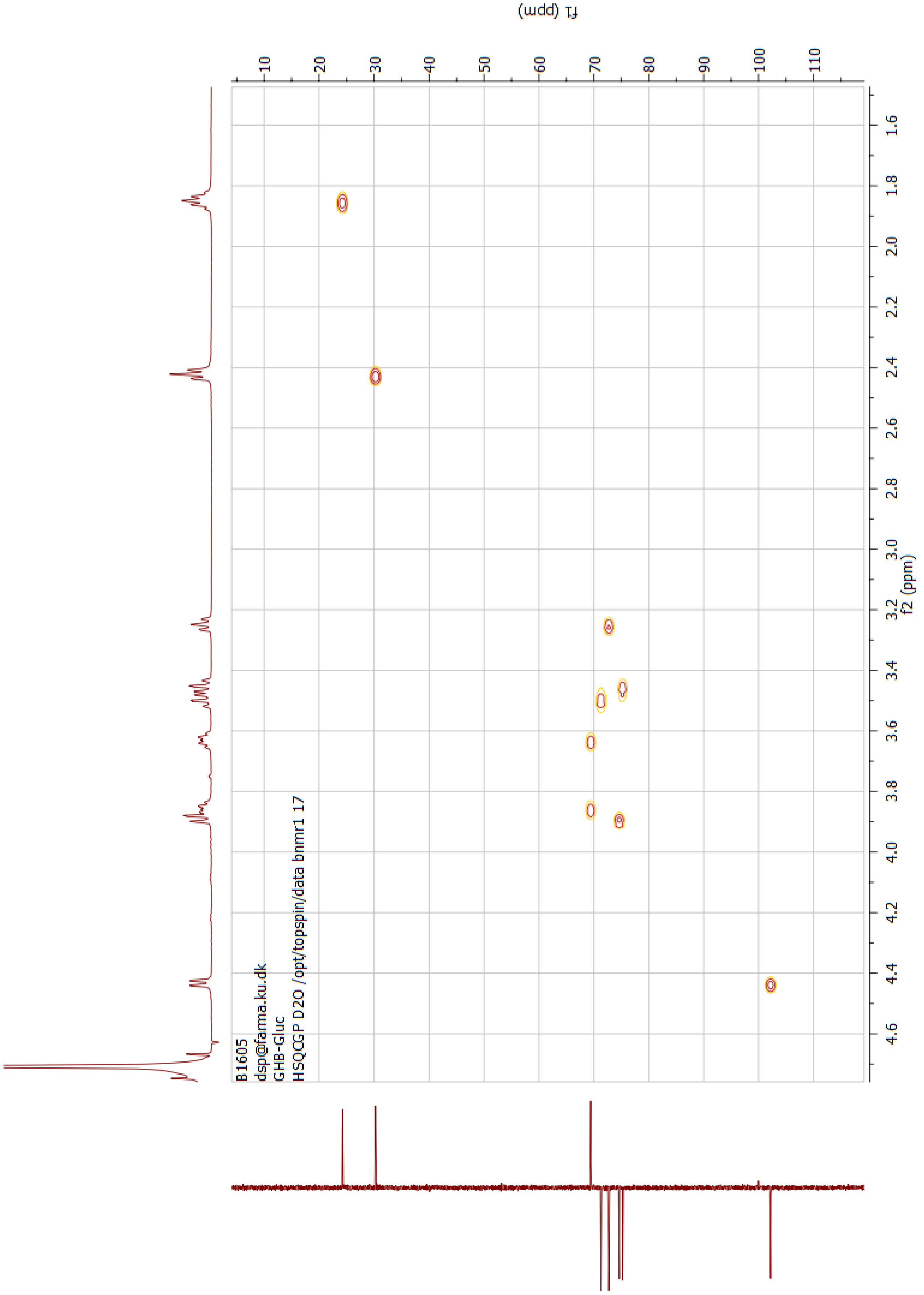

HMBC NMR spectrum of GHB glucuronide 2

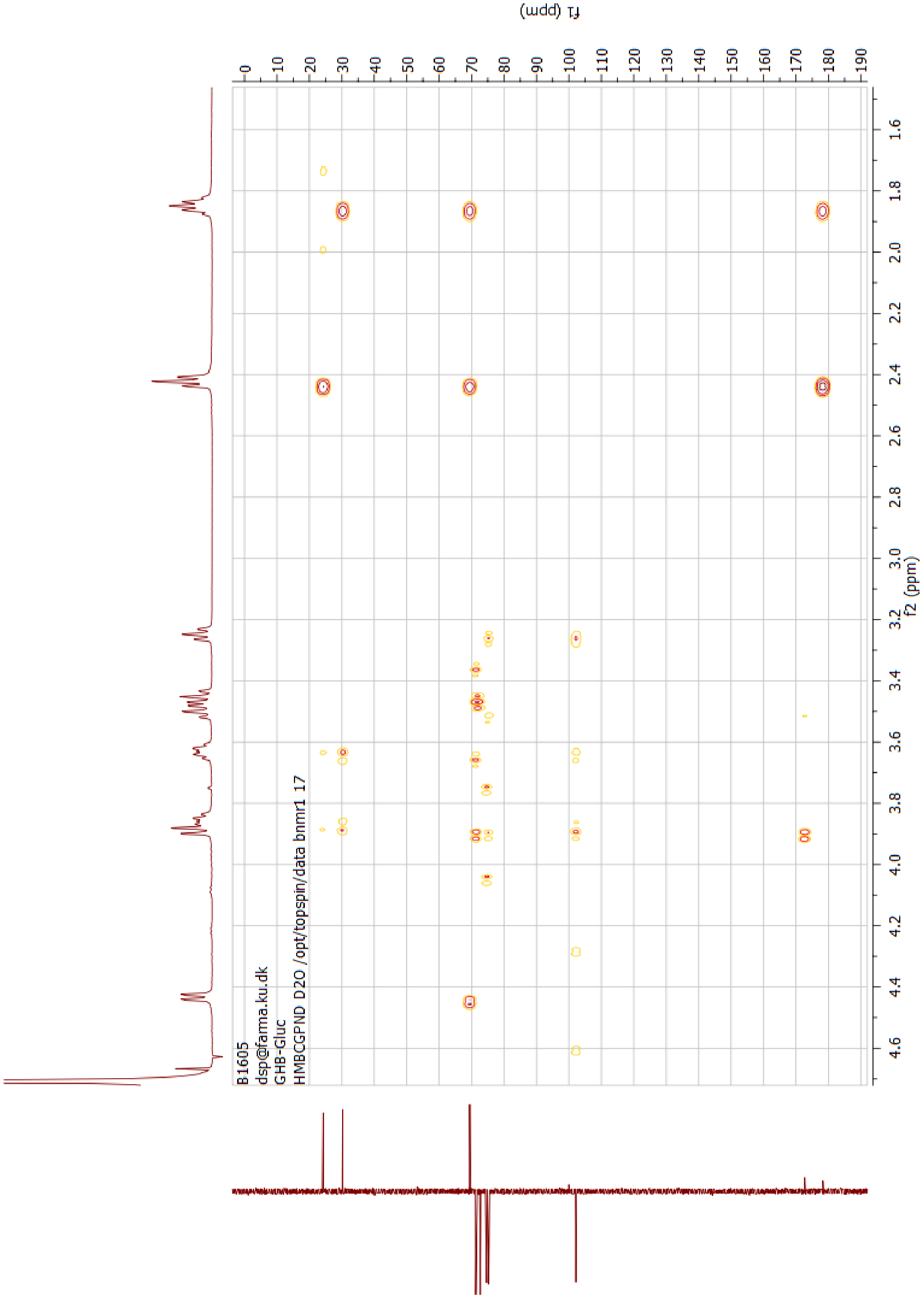

1.2 1D and 2D NMR spectra of glucuronide *d*$_4$-2

^{1}H NMR spectra of GHB glucuronide *d*$_4$-2

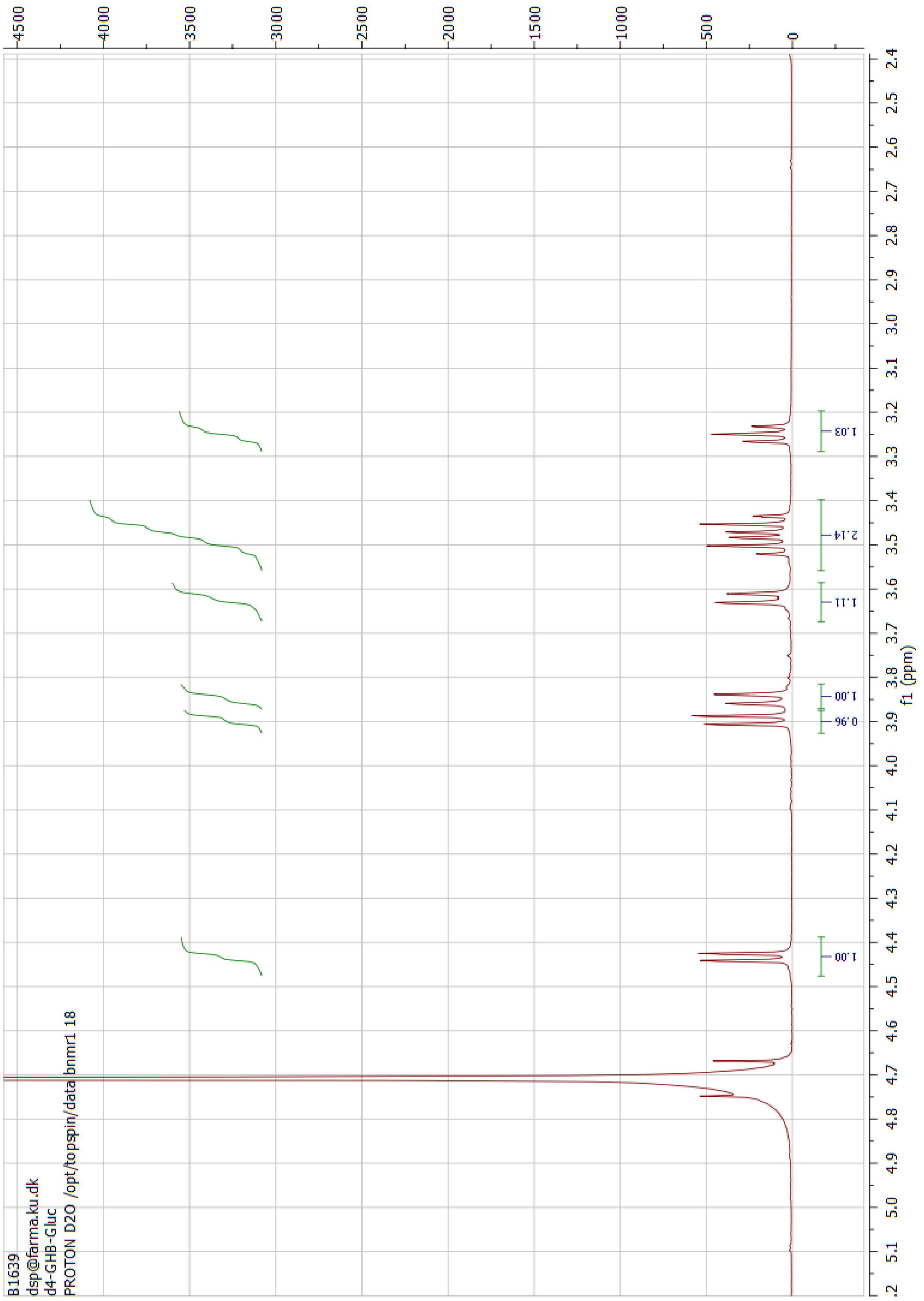

COSY NMR of GHB glucuronide d_4-2

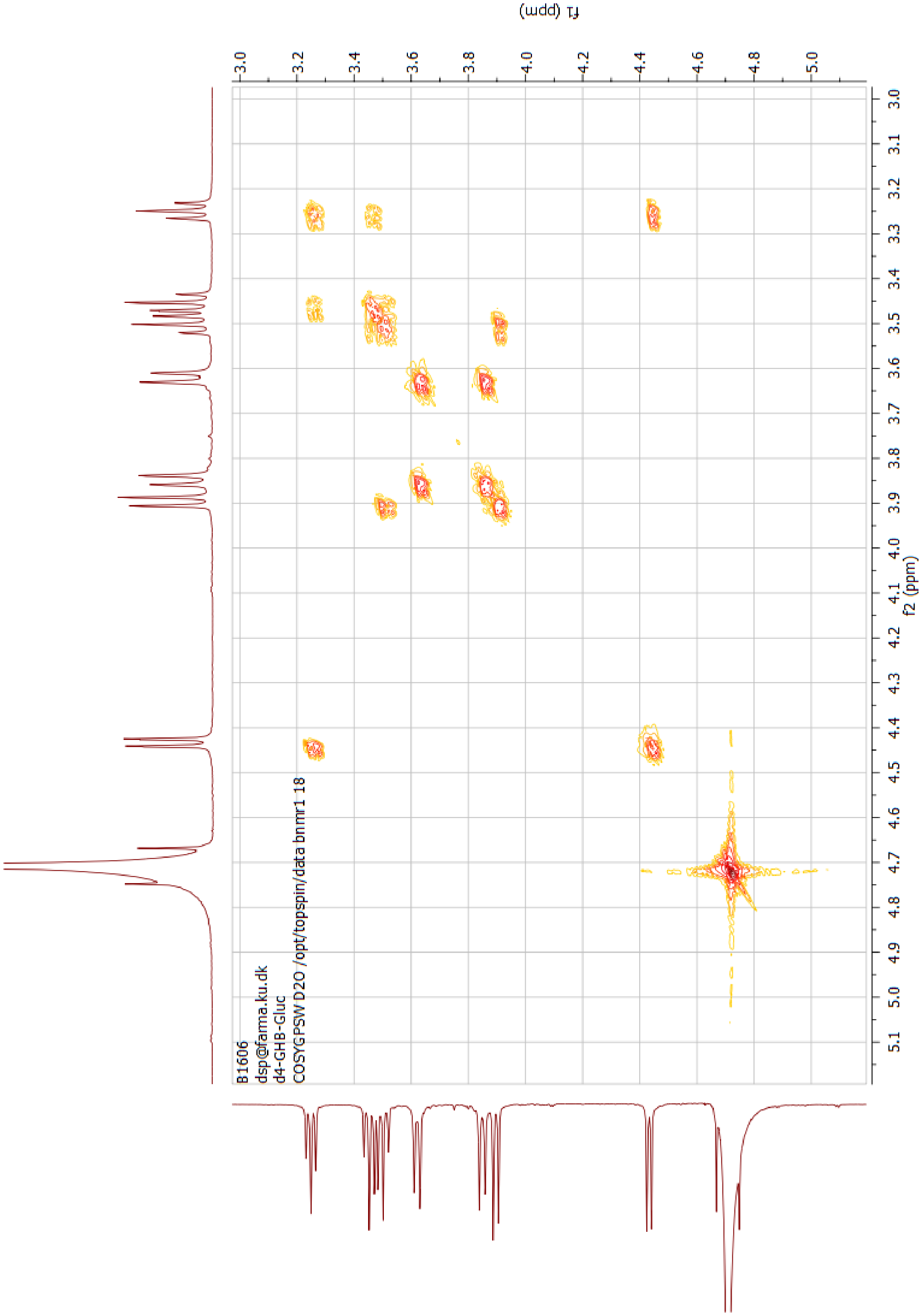

^{13}C NMR of GHB glucuronide *d*$_4$-2

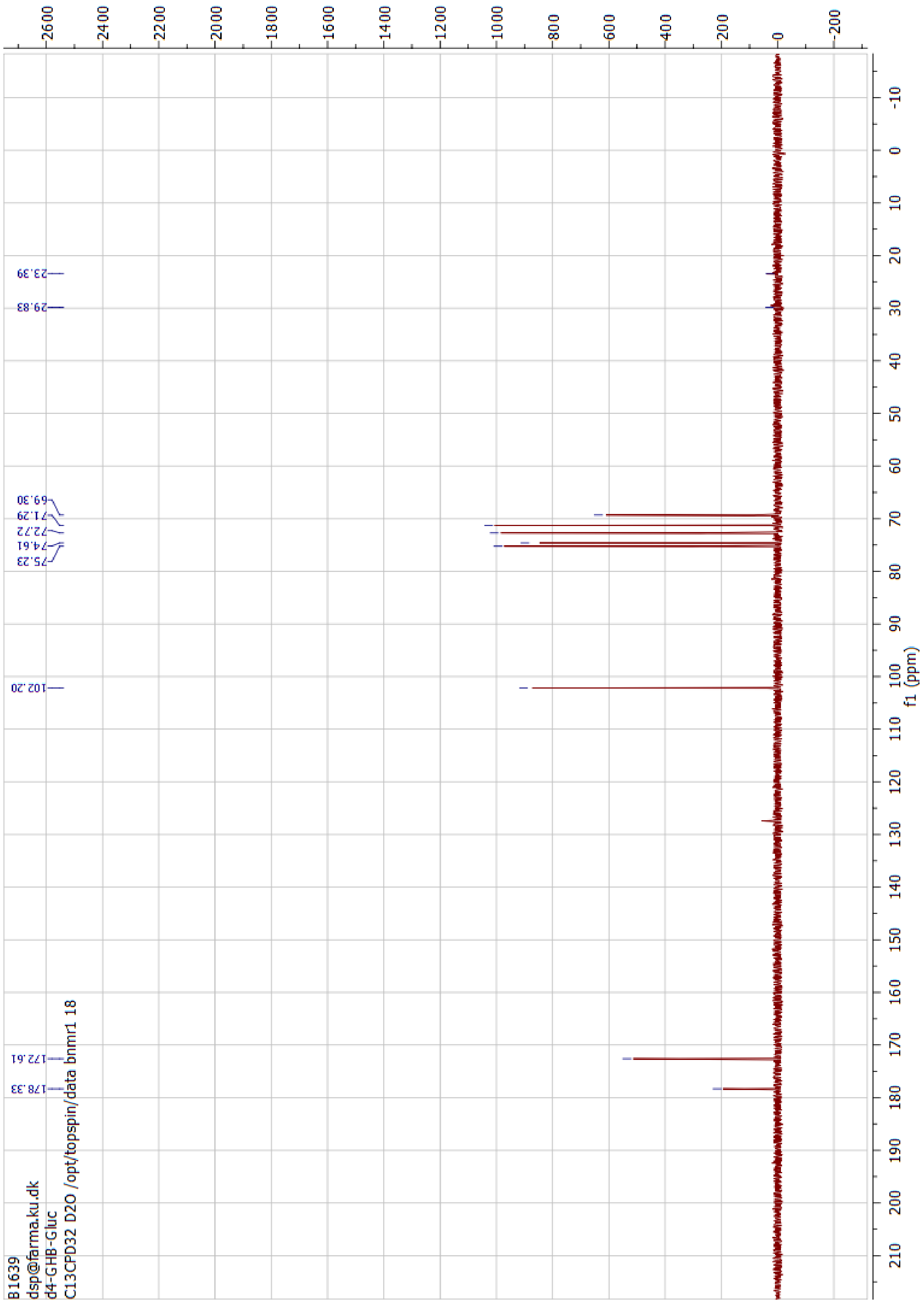

HSQC NMR of GHB glucuronide d_4-2

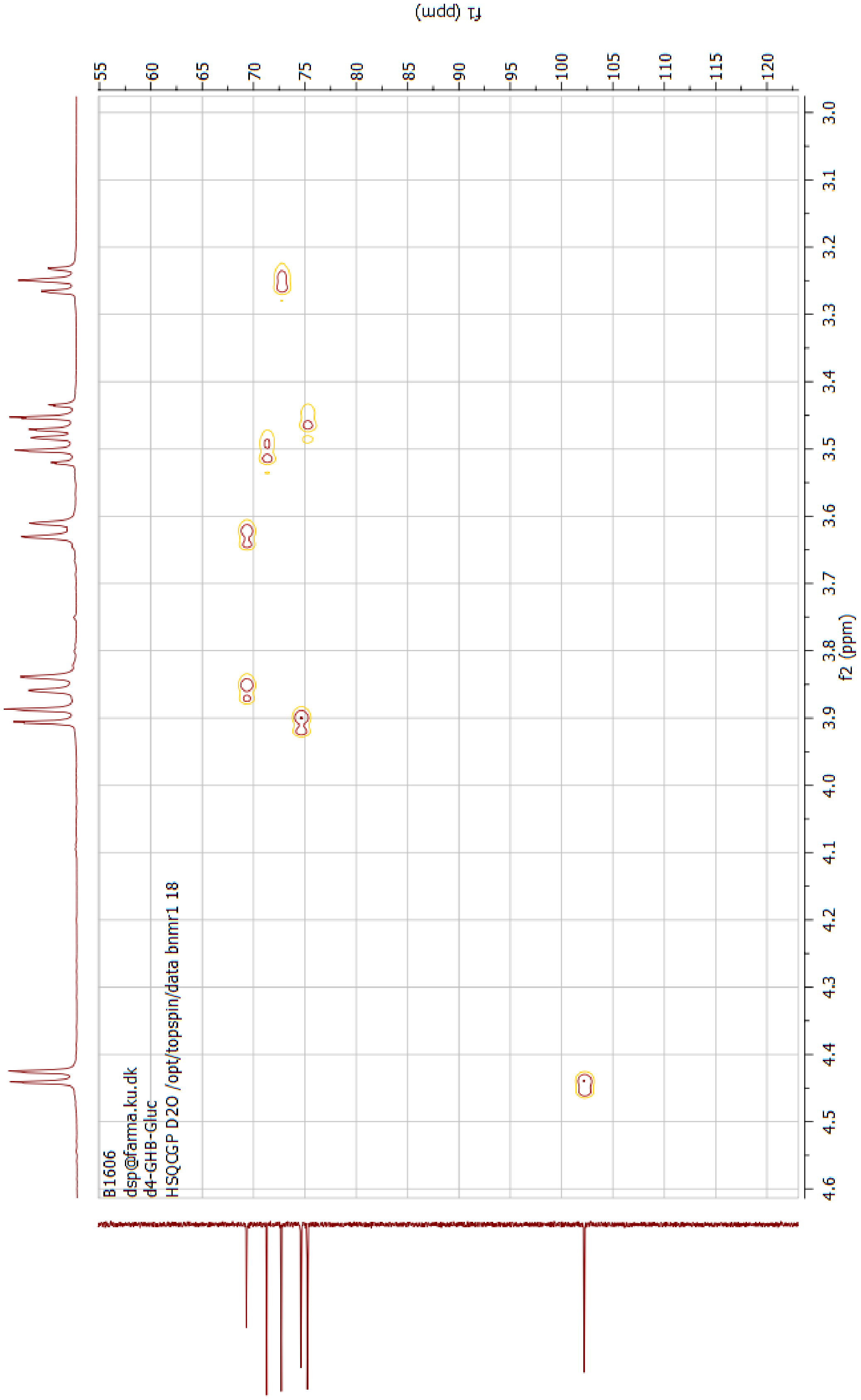

HMBC NMR of GHB glucuronide d_4-2

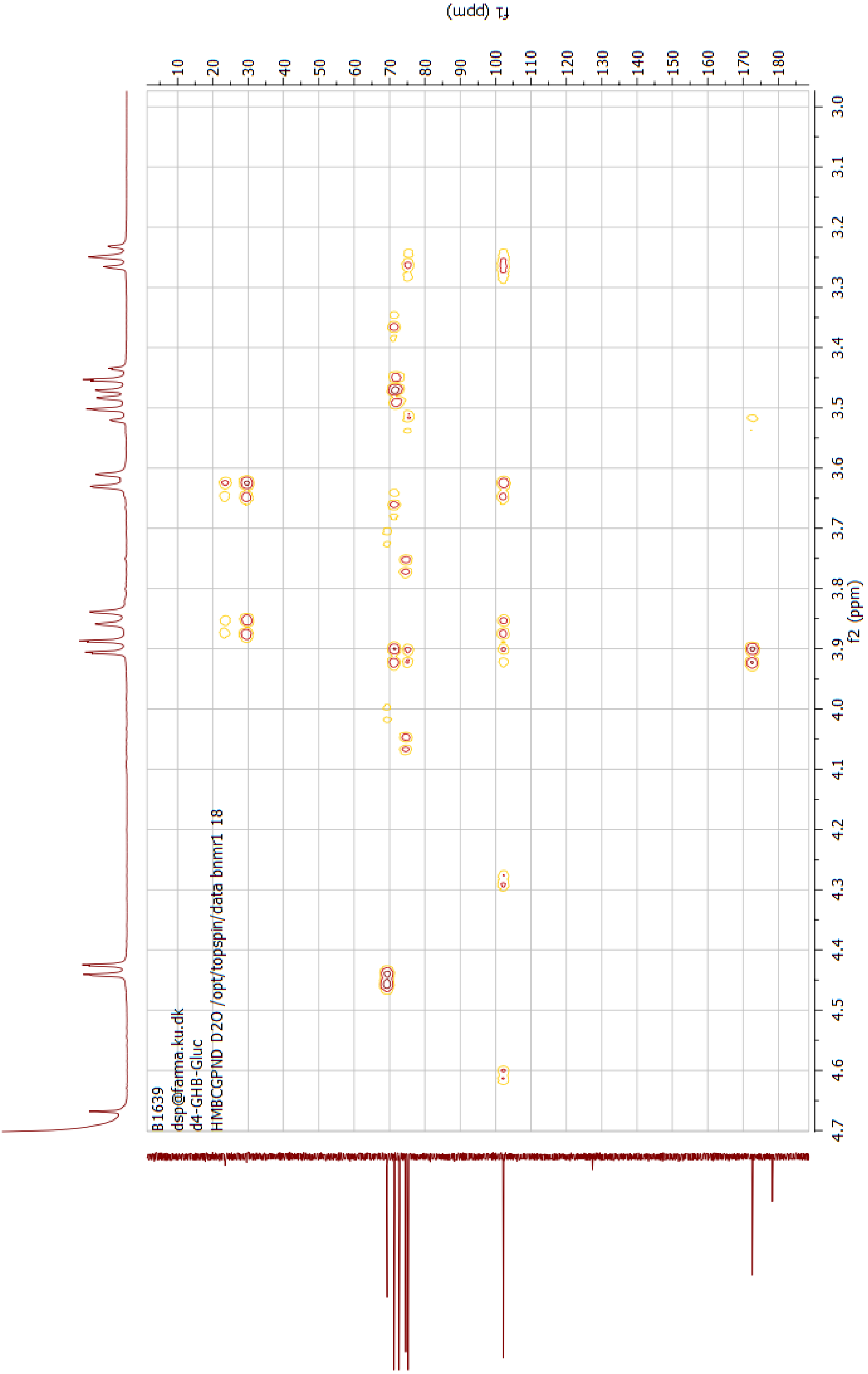

2. NMR stability study for GHB-GLUC (2)

2.1 General NMR experimental

All NMR spectra were recorded on a Bruker 500 MHz Avance III NMR spectrometer. A Watergate-type water-suppression pulse sequence using excitation sculpting with gradients was employed (zgesgp) [1].

Stock solutions:

Buffer A: 10 mM sodium phosphate monobasic (monohydrate) in 90% H_2O/10 D_2O. pH = 4.8.

Buffer B: 10 mM sodium phosphate dibasic (heptahydrate) in 90% H_2O/10 D_2O. pH = 9.0.

Buffers were prepared by dissolving the appropriate salt (**A**: 1.38 g and **B**: 2.68 g) in 10 mL D_2O and adjusting the volume to 100 mL with mQ water.

pH was determined using a Radiometer Copenhagen PHM92 pH meter after calibration using IUPAC calibration solutions pH 4.005 and 10.012 from Radiometer Analytical.

Internal standard: 0.1 M *t*-BuOH in H_2O. 0.74 g *t*-BuOH was dissolved in H_2O to give a final volume of 100 mL.

NMR buffer A with internal standard: Prepared by taking 800 µL internal standard solution and adding Buffer A to give a final volume of 20 mL (final *t*-BuOH concentration 4 µM).

NMR buffer B with internal standard: Prepared by taking 800 µL internal standard solution and adding Buffer B to give a final volume of 20 mL (final *t*-BuOH concentration 4 µM).

Determination of integration accuracy:

Watergate-type water suppression during NMR experiments can affect integration accuracy. Hence the NMR method was validated by measuring known concentrations of GHB and comparing them to an internal standard (*t*-BuOH). Three samples with increasing

concentration of *t*-BuOH relative to GHB were analyzed by ^{1}H NMR. Integration in ^{1}H NMR spectra was accurate for all three GHB methylene groups over the examined concentration range (deviation < ± 1%).

<u>NMR samples for determining integration accuracy:</u>

GHB (3.0 mg) was dissolved in NMR buffer A with internal standard (1.0 mL) to give NMR sample C = 1.

NMR sample C = 1 (0.5 mL) was diluted with NMR buffer with internal standard (0.5 mL) to give NMR sample C = 2.

NMR sample C = 2 (0.5 mL) was diluted with NMR buffer with internal standard (0.5 mL) to give NMR sample C = 4.

NMR Samples

GHB-GLUC (1 mg, 3.6 μmol) was dissolved in NMR buffer A with internal standard (0.6 mL).

GHB-GLUC (1 mg, 3.6 μmol) was dissolved in NMR buffer B with internal standard (0.6 mL).

The stability of GHB-GLUC in both buffer systems was evaluated over time at 18, 60 and 90 °C.

<u>Reference spectra (^{1}H and ^{13}C):</u>

a) *t*-BuOH (12 mg) in 10% D_2O in H_2O (1.0 mL).
b) γ-Butyrolactone (2 mg) in NMR buffer A (1.0 mL).
c) γ-Hydroxybutyric acid in NMR buffer A. Integration experiment at [*C*] = 1, 2 and 4 (vide supra).
d) β-D-Glucuronic acid (2 mg) in NMR buffer A (1.0 mL).

2.2 NMR stability study ^{1}H NMR spectra

^{1}H NMR of 2 in NMR buffer A with internal standard at 18 °C (0–120 h)

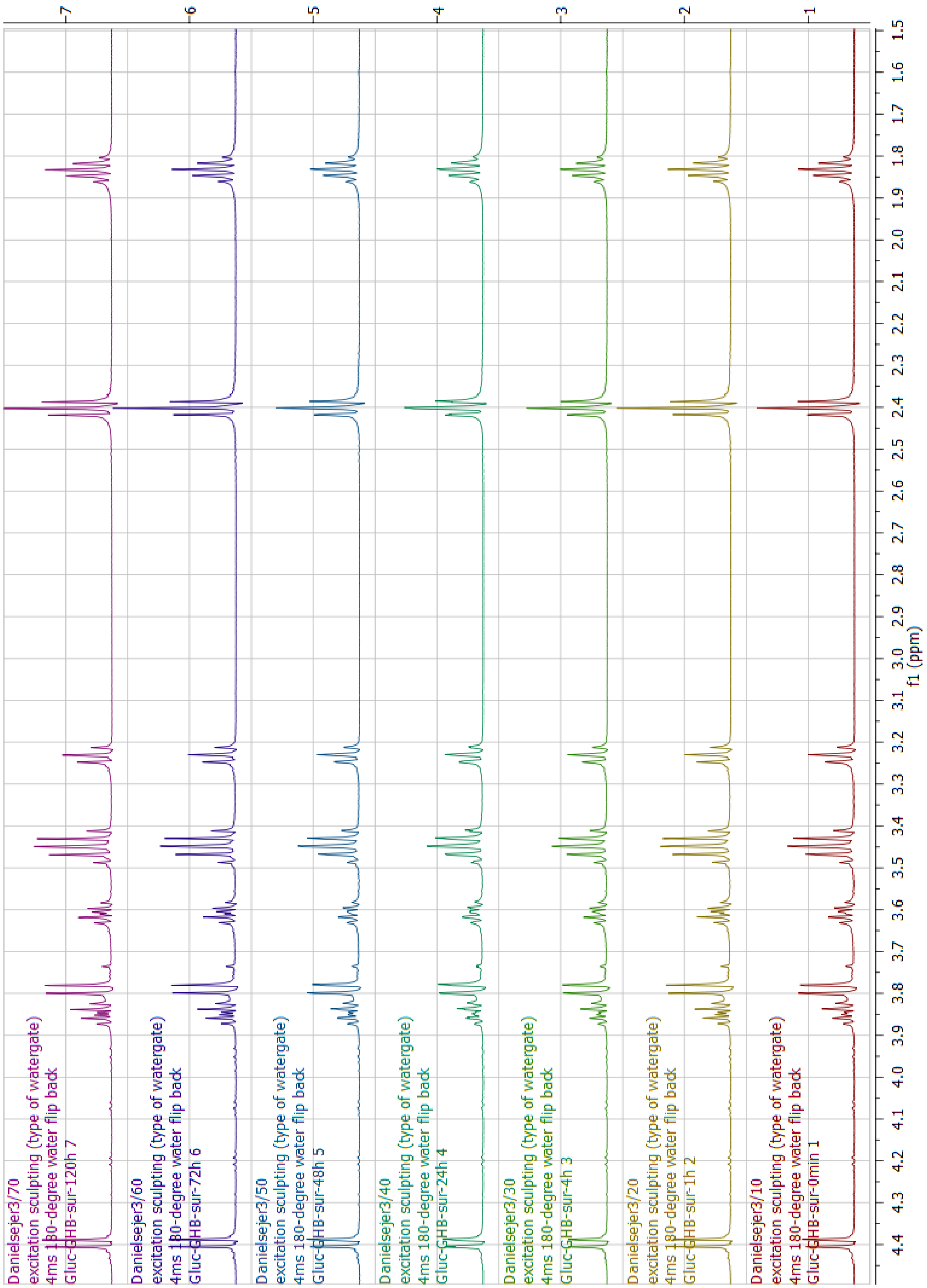

^{1}H NMR of 2 in NMR buffer A with internal standard at 60 °C (0.5–24 h) and 90 °C (1–72 h)

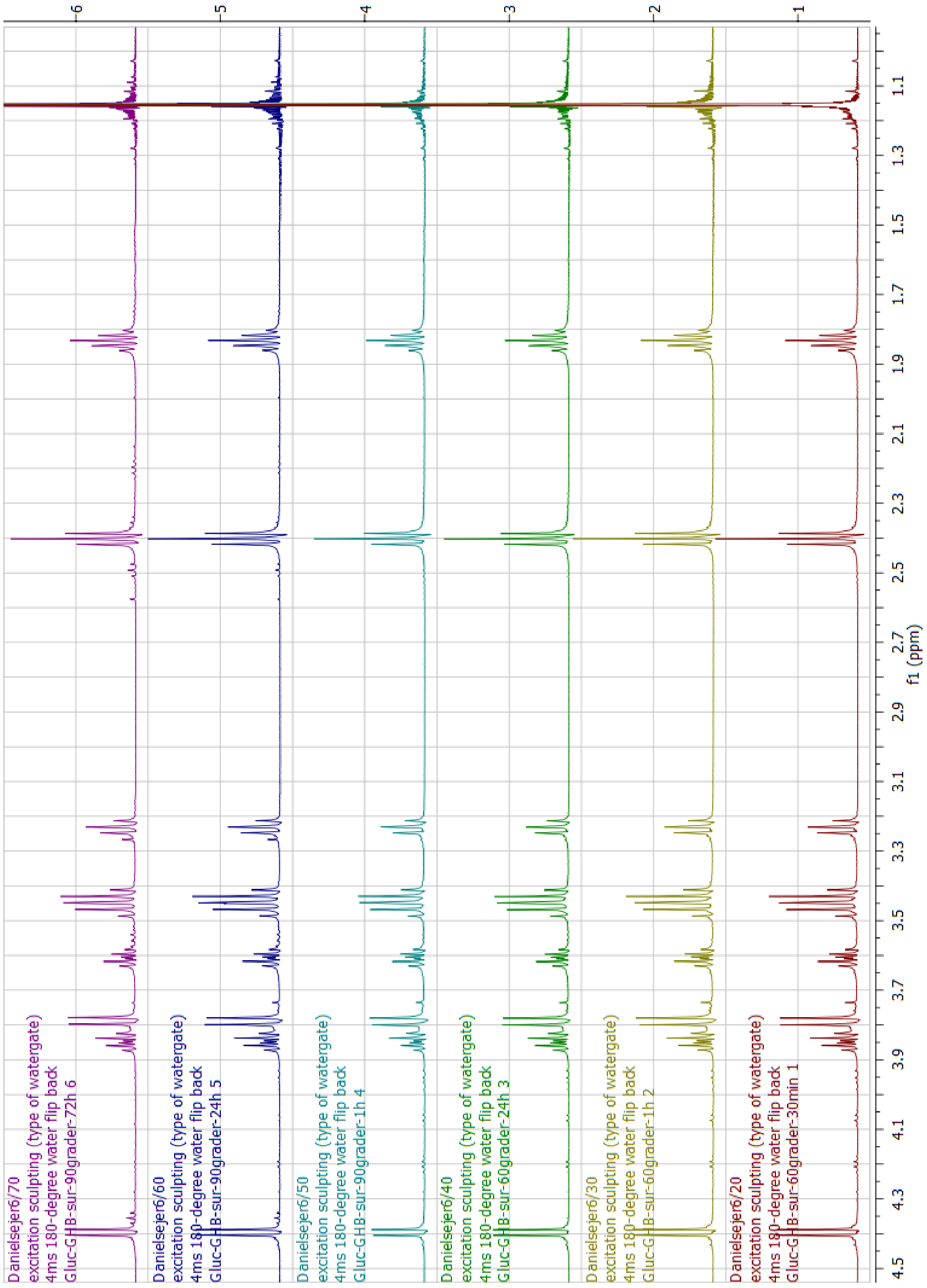

¹H NMR of 2 in NMR buffer B with internal standard at 60 °C (0.5–24 h) and 90 °C (1–72 h)

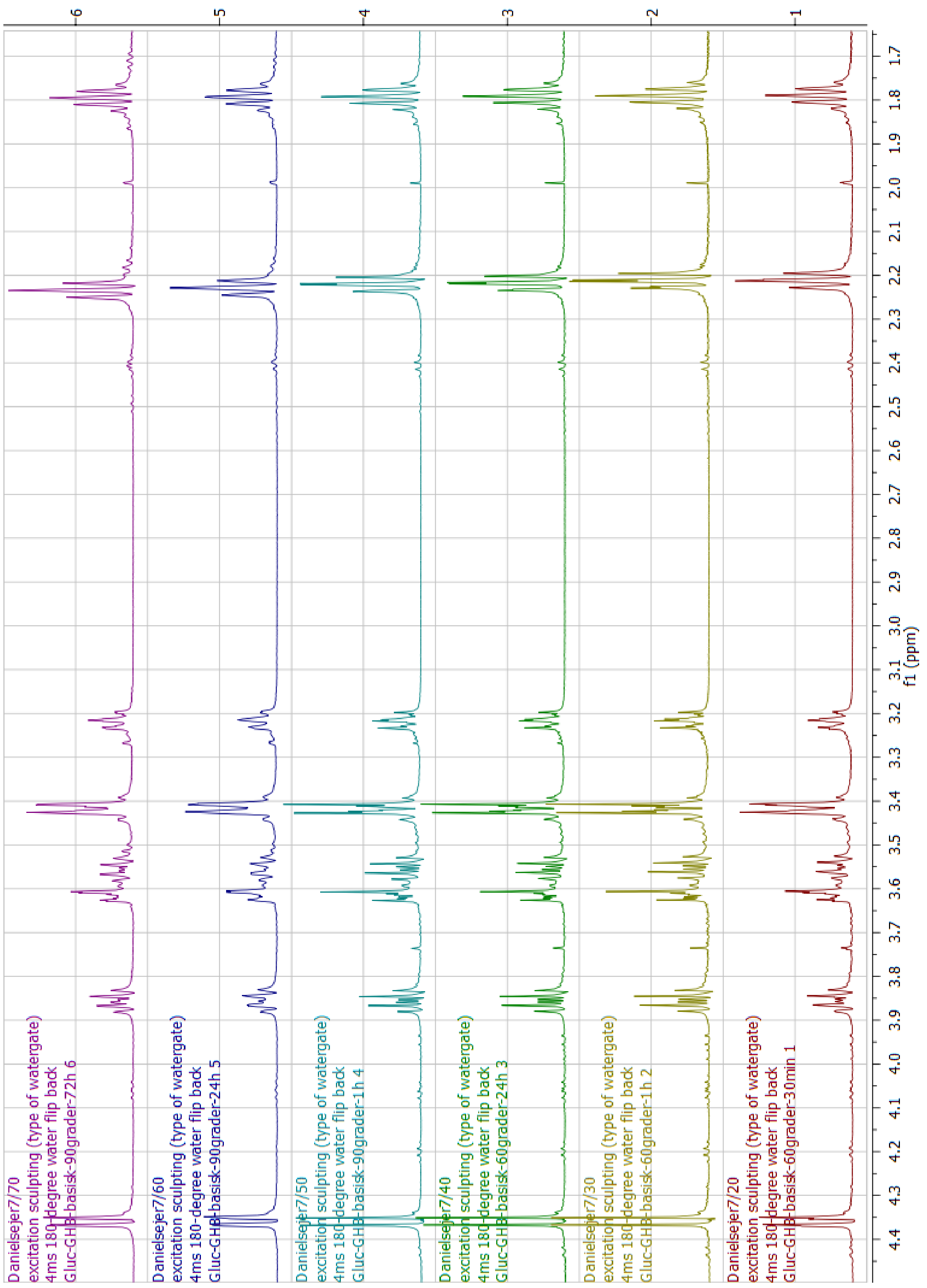

2.3 NMR reference spectra

^1H NMR of *t*-BuOH in 10% D_2O in H_2O

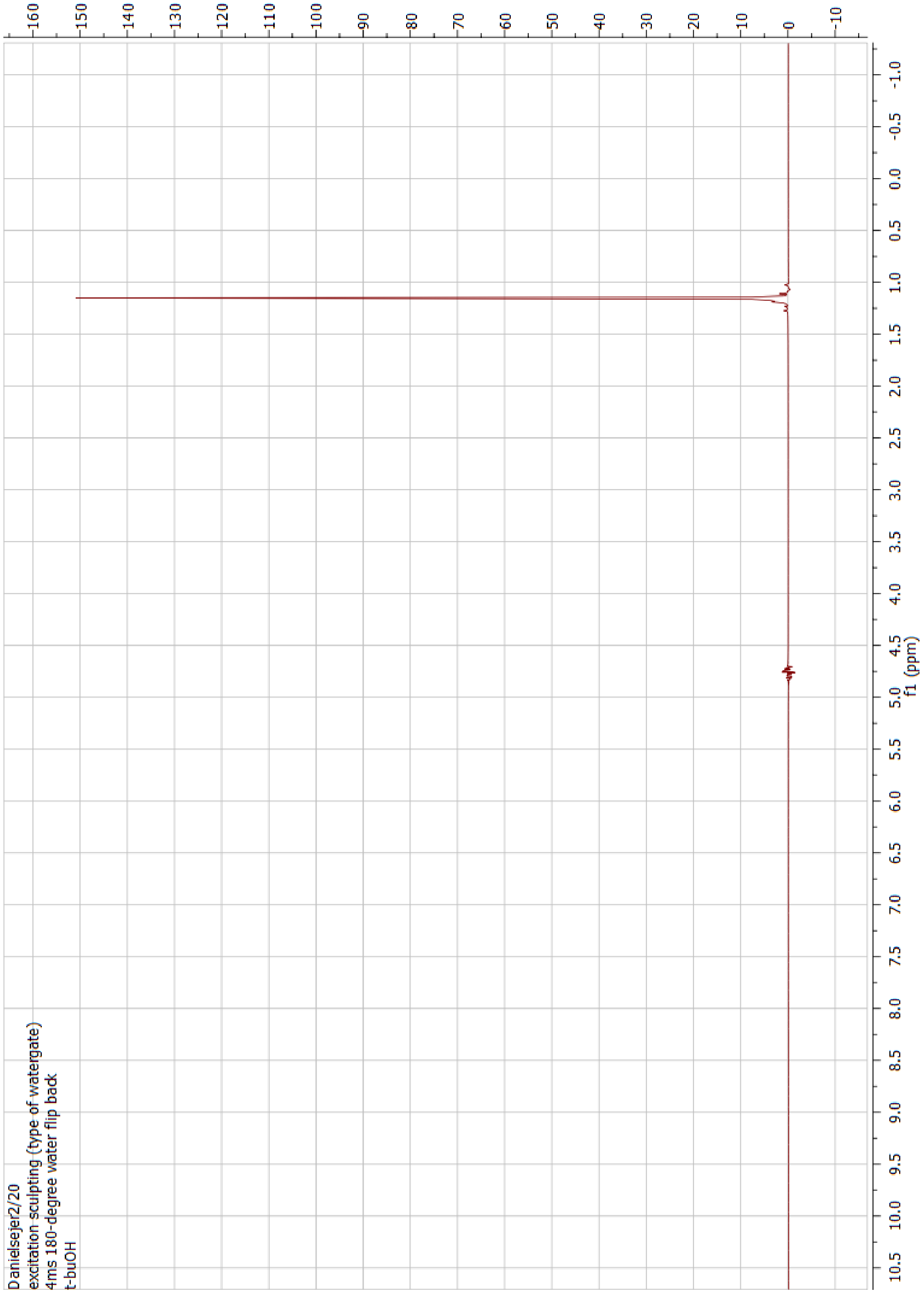

^{13}C APT NMR of *t*-BuOH in 10% D_2O in H_2O

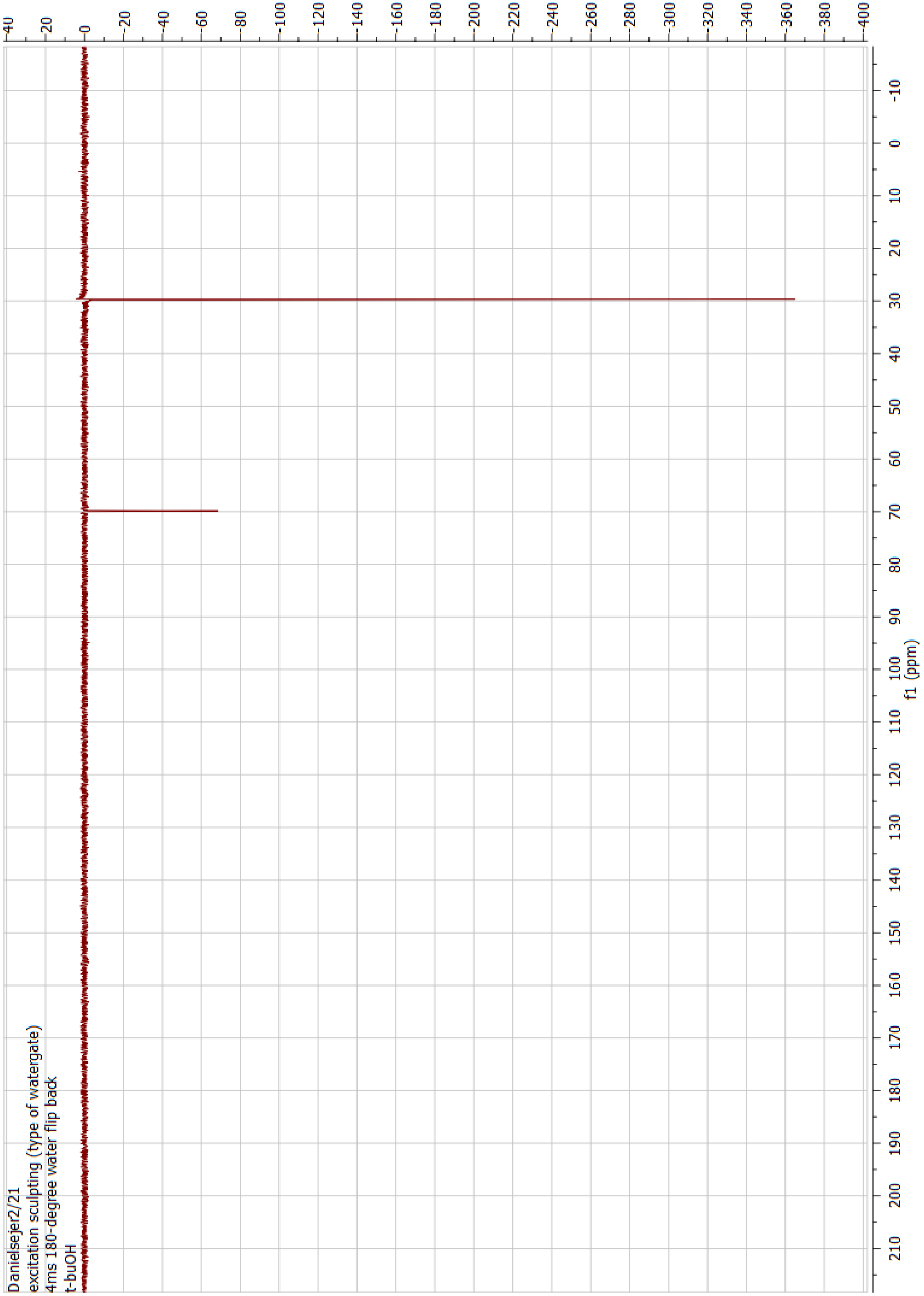

^{1}H NMR of γ-butyrolactone (GBL) in NMR buffer A

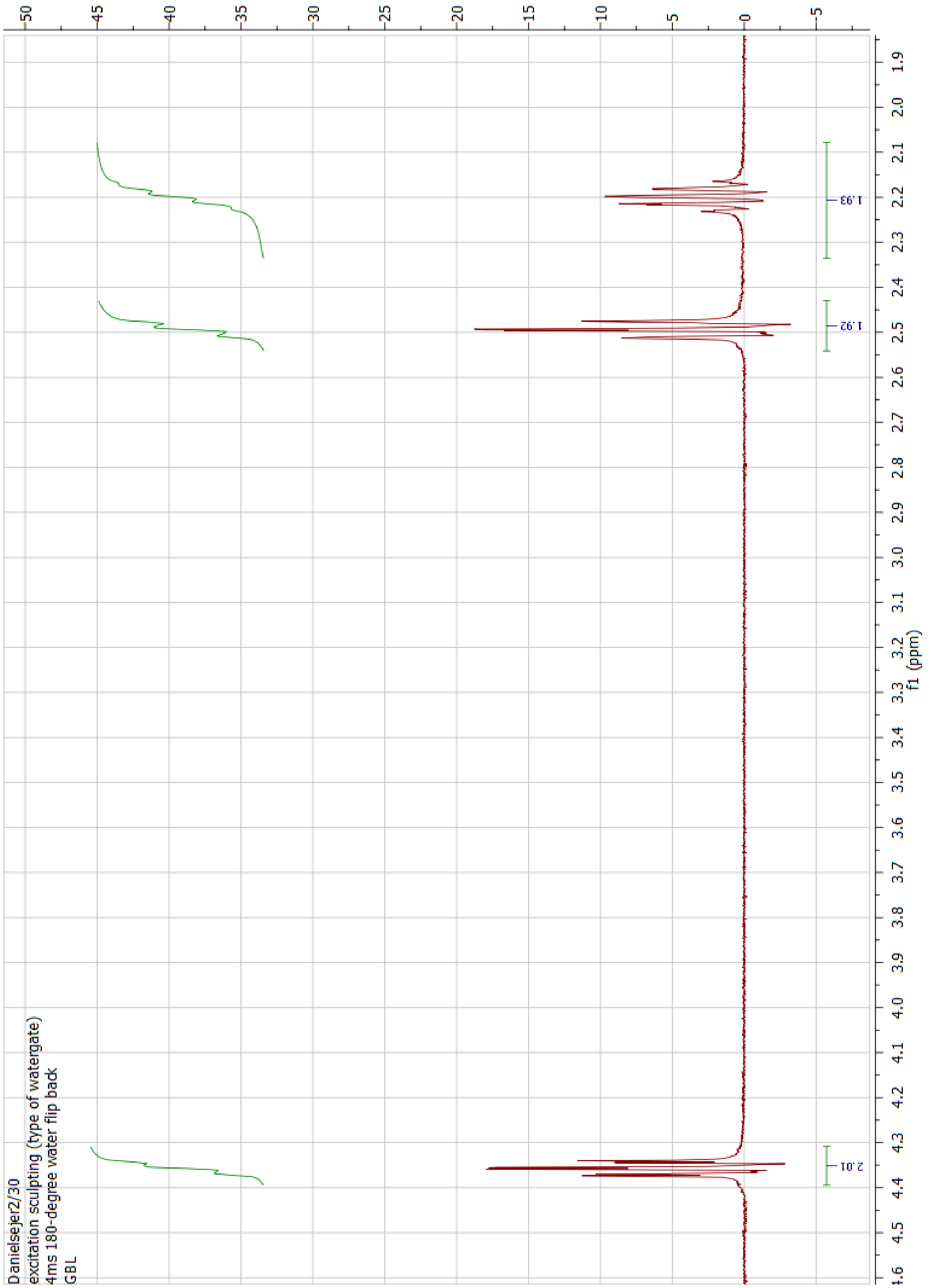

^{13}C NMR of γ-butyrolactone (GBL) in NMR buffer A

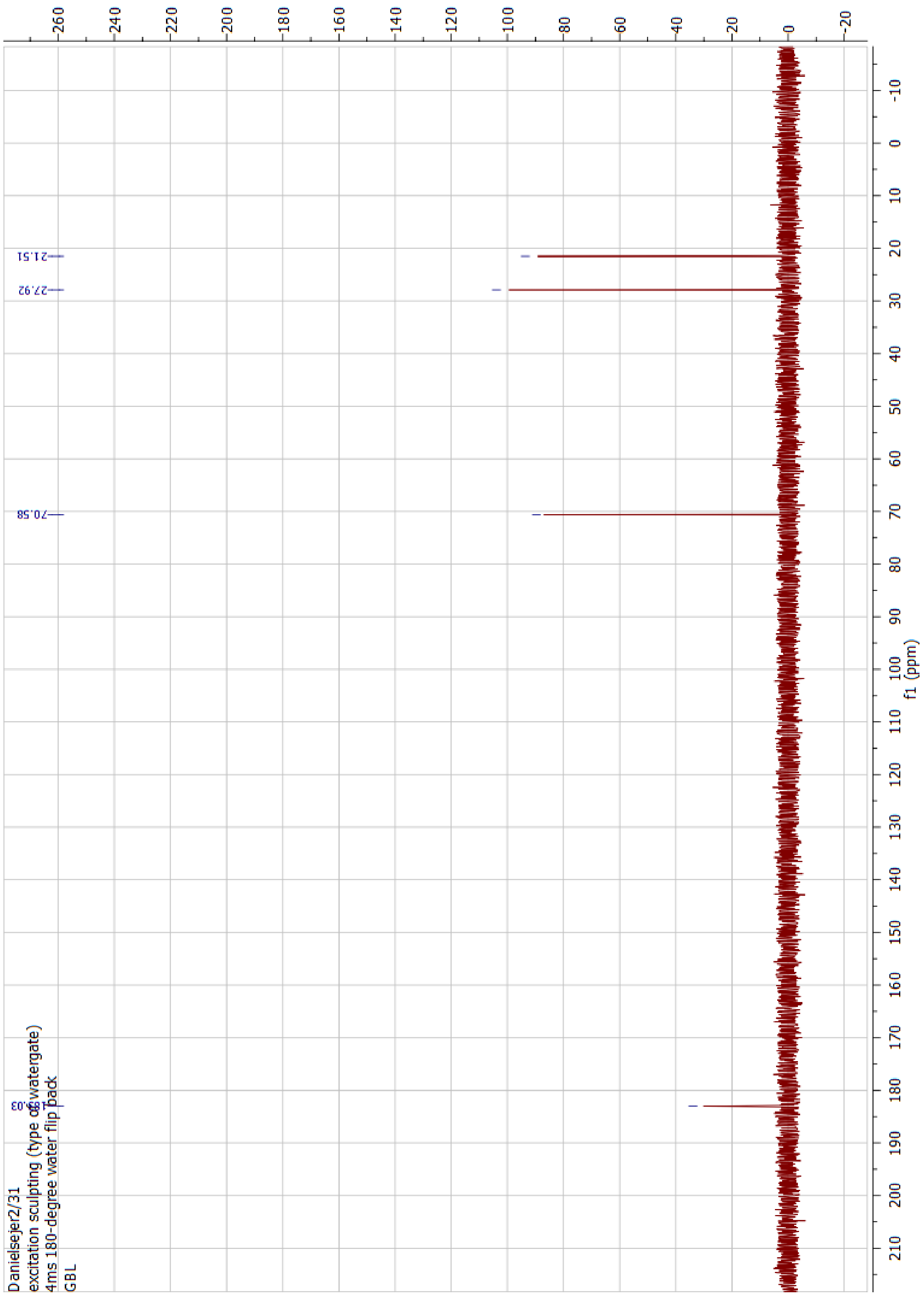

^{1}H NMR of γ-hydroxybutyric acid (GHB) in NMR buffer A, [*C*] = 1

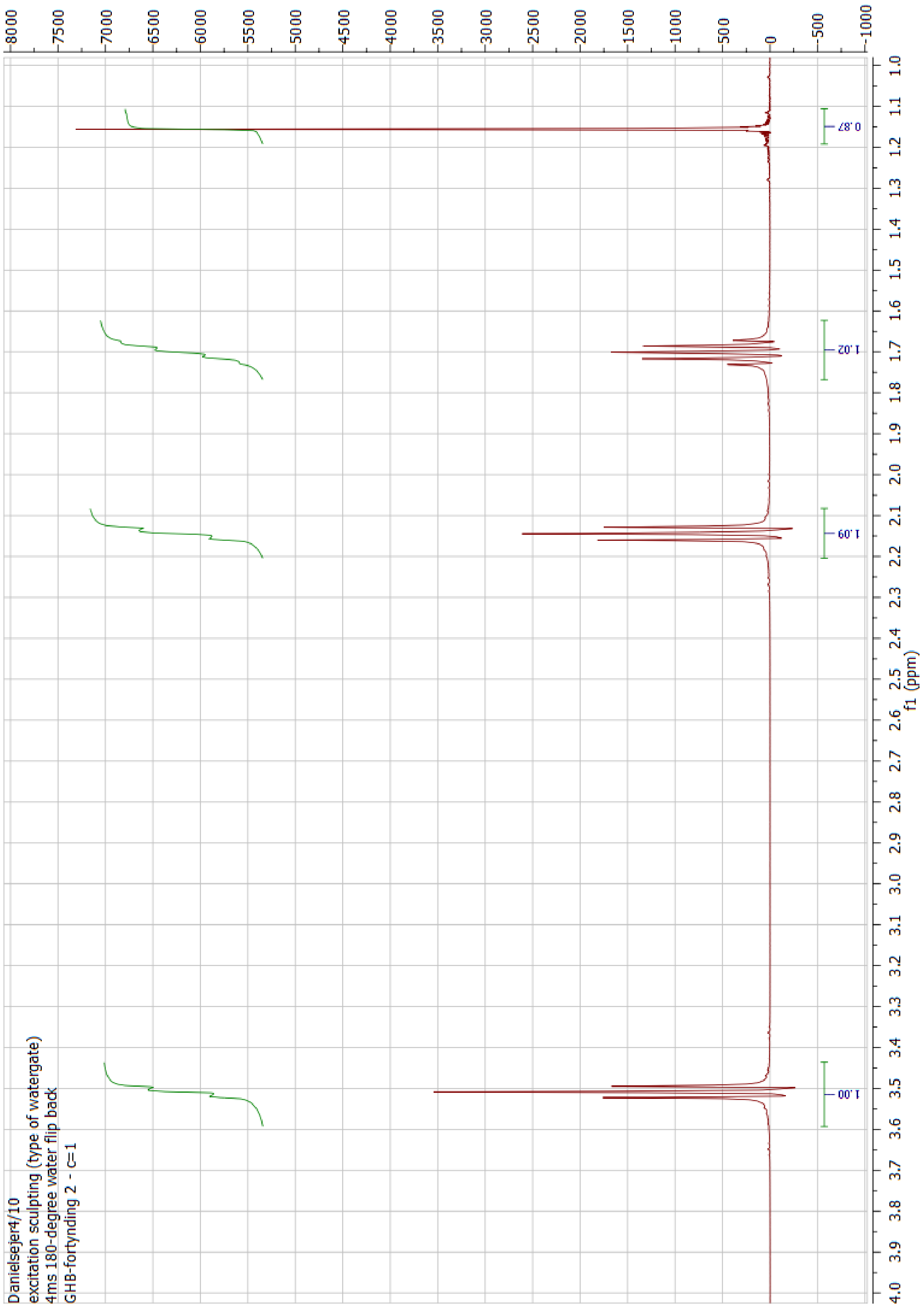

^{1}H NMR of γ-hydroxybutyric acid (GHB) in NMR buffer A, [*C*] = 2

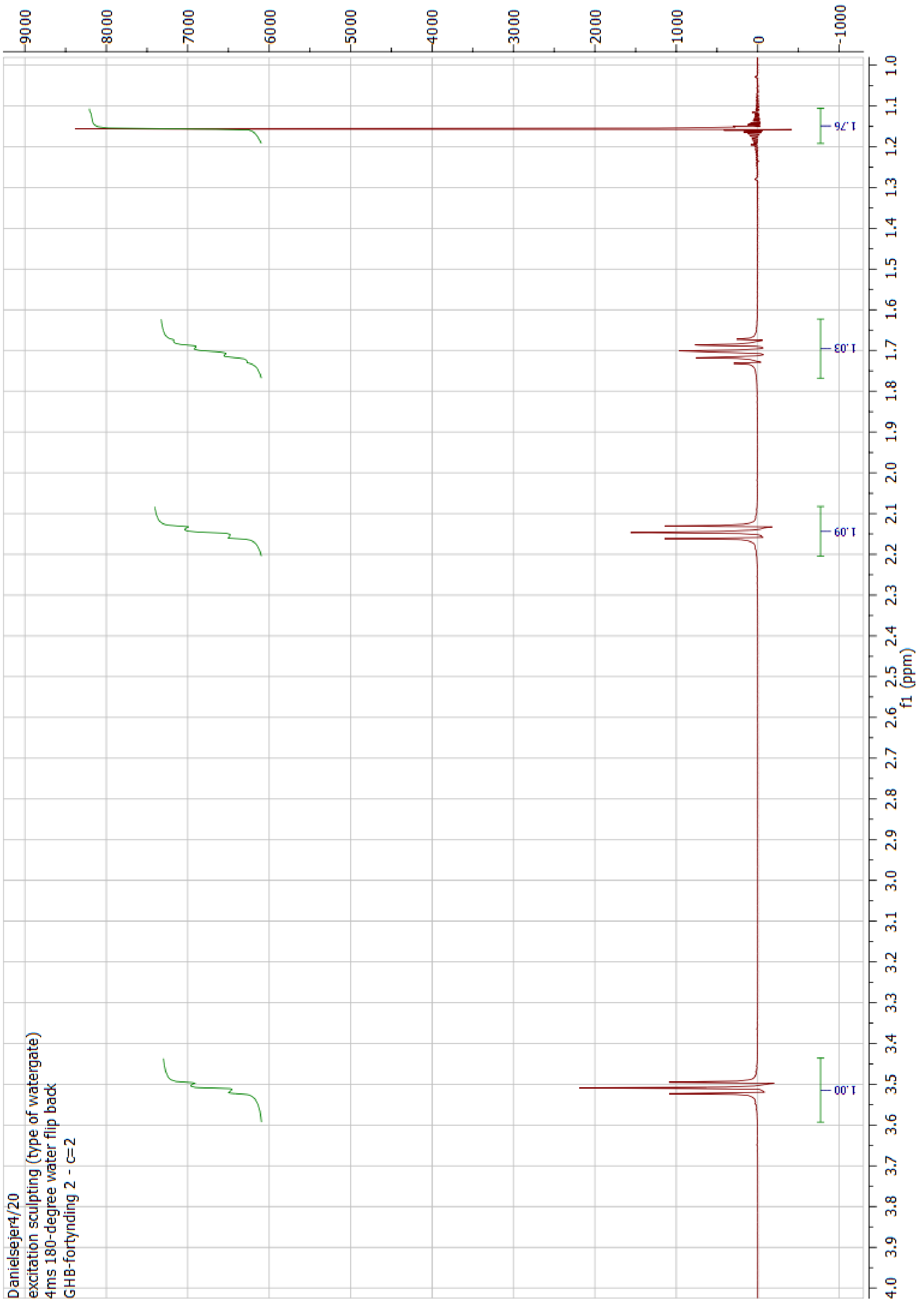

^{1}H NMR of γ-hydroxybutyric acid (GHB) in NMR buffer A, [*C*] = 4

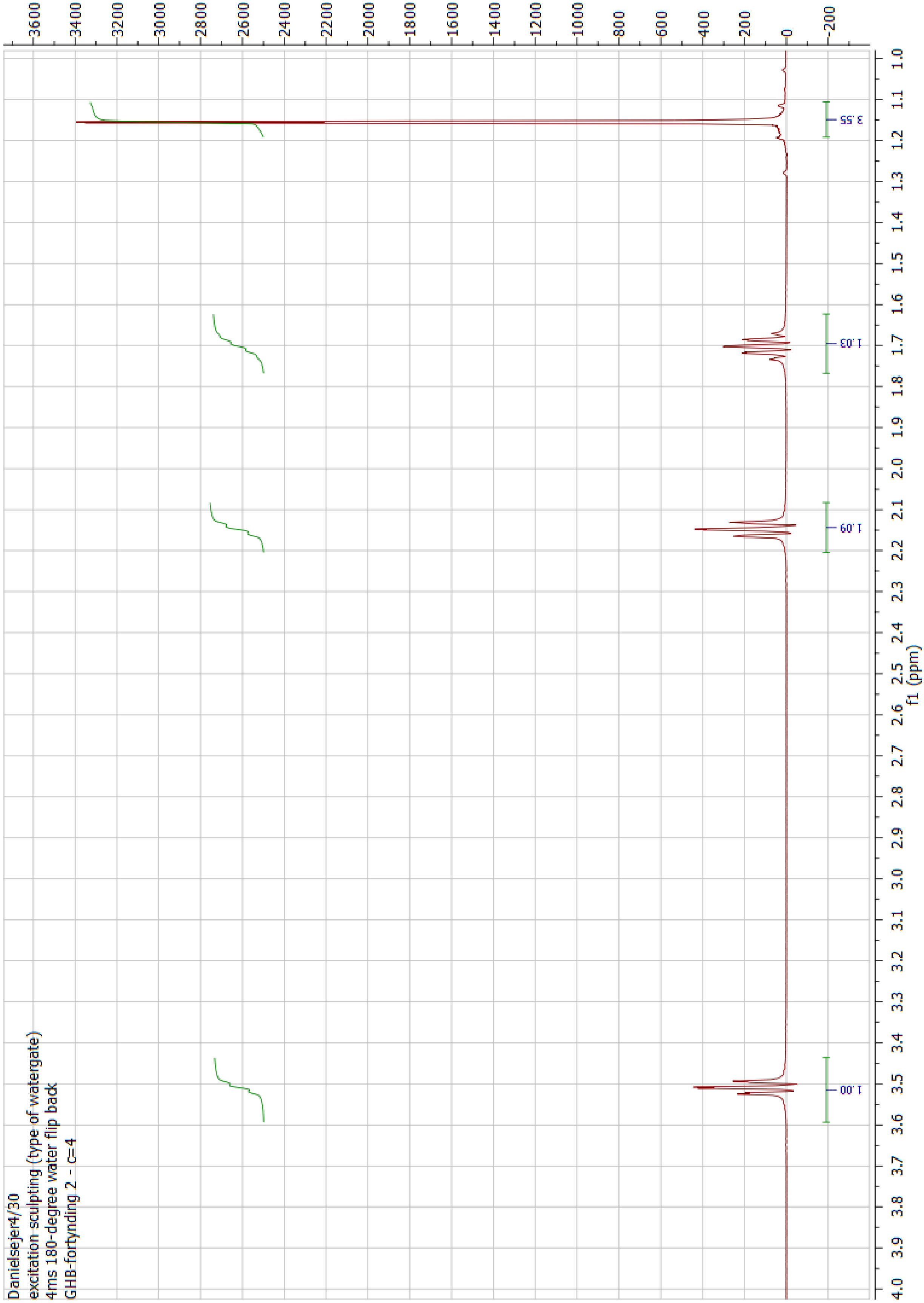

^{1}H NMR of β-D-glucuronic acid in NMR buffer A

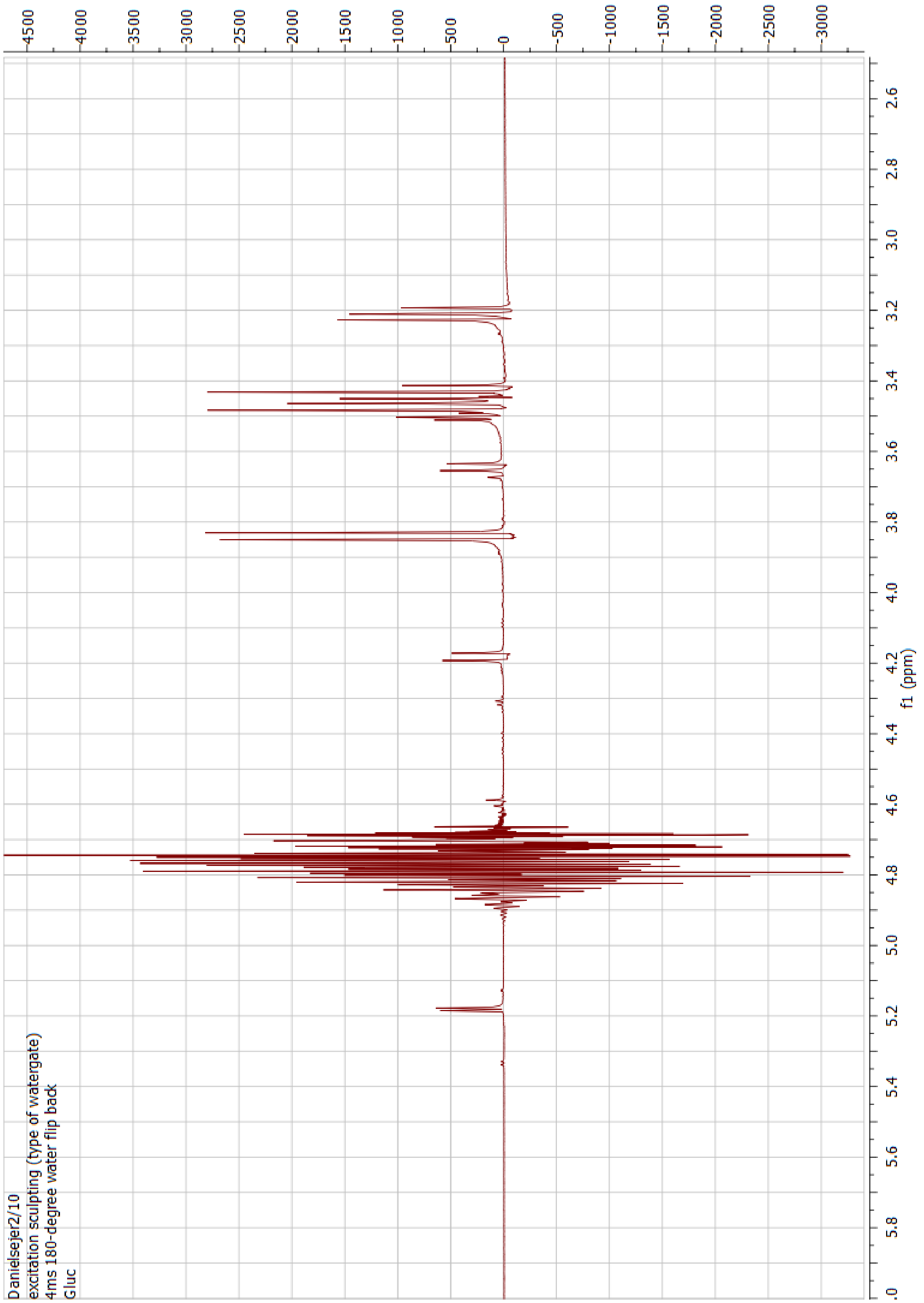

^{13}C NMR of β-D-glucuronic acid in NMR buffer A

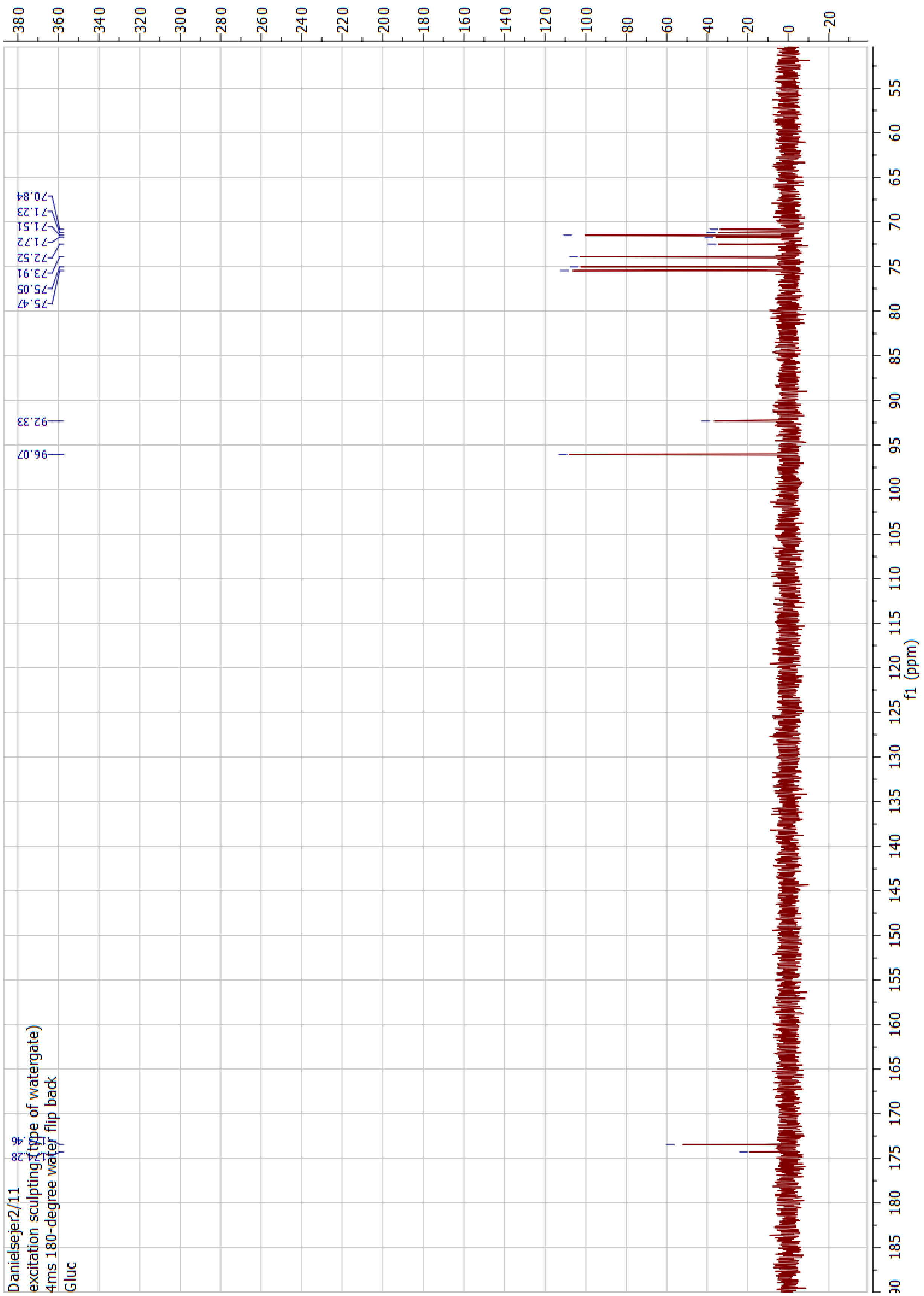

Reference

[1] Hwang, T. L.; Shaka, A. J. *J. Mag.Reson. Series A* **1995,** *112,* 275–279
